# Association of Aortic Stiffness and Cognitive Decline: A Systematic Review and Meta-Analysis

**DOI:** 10.3389/fnagi.2021.680205

**Published:** 2021-06-24

**Authors:** Qian Liu, Jinghuan Fang, Chaohua Cui, Shuju Dong, Lijie Gao, Jiajia Bao, Yanbo Li, Mengmeng Ma, Ning Chen, Li He

**Affiliations:** Department of Neurology, West China Hospital, Sichuan University, Chengdu, China

**Keywords:** aortic stiffness, pulse wave velocity, cognitive impairment, vascular dementia, aging

## Abstract

**Background:** Increased aortic stiffness has been found to be associated with cognitive function decline, but the evidence is still under debate. It is of great significance to elucidate the evidence in this debate to help make primary prevention decisions to slow cognitive decline in our routine clinical practice.

**Methods:** Electronic databases of PubMed, EMBASE, and Cochrane Library were systematically searched to identify peer-reviewed articles published in English from January 1, 1986, to March 16, 2020, that reported the association between aortic stiffness and cognitive function. Studies that reported the association between aortic pulse wave velocity (PWV) and cognitive function, cognitive impairment, and dementia were included in the analysis.

**Results:** Thirty-nine studies were included in the qualitative analysis, and 29 studies were included in the quantitative analysis. The aortic PWV was inversely associated with memory and processing speed in the cross-sectional analysis. In the longitudinal analysis, the high category of aortic PWV was 44% increased risk of cognitive impairment (OR 1.44; 95% CI 1.24–1.85) compared with low PWV, and the risk of cognitive impairment increased 3.9% (OR 1.039; 95% CI 1.005–1.073) per 1 m/s increase in aortic PWV. Besides, meta-regression analysis showed that age significantly increased the association between high aortic PWV and cognitive impairment risk.

**Conclusion:** Aortic stiffness measured by aortic PWV was inversely associated with memory and processing speed and could be an independent predictor for cognitive impairment, especially for older individuals.

## Introduction

With the population aging, an increasing number of older adults suffer from cognitive impairment and dementia, which substantially reduce the quality of life in the elderly and bring a substantial medical burden to their family and the whole society (Langa and Levine, 2014). It is of great significance to recognize the risk factors to prevent cognitive impairment and dementia (Livingston et al., 2017).

In recent years, through the growing investigations and the more in-depth understanding of aortic stiffness, it was found that aortic stiffness is not only related to increased risk of cardiovascular diseases and related mortality (Vlachopoulos et al., 2010) but also involved in the aging changes of brain and cognitive function (Vlachopoulos et al., 2010; Zeki Al Hazzouri et al., 2013; Yukutake et al., 2015; Iulita et al., 2018; Rouch et al., 2018). With advancing age, the aortic vessel wall's elastic fibers are gradually reduced and replaced by collagen fibers or deposition of calcification, which impairs elastic aorta's elasticity and causes aortic stiffness (Thorin-Trescases and Thorin, 2016). The stiffening and loss of recoil in the aorta would transmit excessive and damaging pulsatile load to the peripheral arteries of body organs. Theoretically, the brain is more susceptible to pulsatile damage due to its low-resistance and high-flow characteristics (Thorin-Trescases and Thorin, 2016; Iulita et al., 2018). Aortic stiffness was reported to be closely associated with cerebral structural changes, primarily the cerebral small vessel disease and brain atrophy (Mitchell et al., 2011; Webb et al., 2012; van Sloten et al., 2015, 2016). There have been studies that focus on the relationship between aortic stiffness and cognitive function. However, their results were inconsistent (Poels et al., 2007; Singer et al., 2013; van Sloten et al., 2015).

Among various pulse wave velocity (PWV) measurements for aortic stiffness, carotid-femoral PWV (cfPWV) that measure the PWV along the aortic and aortoiliac pathways is the recommended gold-standard non-invasive technique to assess aortic stiffness because of its reliability and feasibility, which is highly related with magnetic resonance imaging (MRI) directly measuring PWV (Laurent et al., 2006; Boutouyrie et al., 2014). While brachial-ankle PWV (baPWV) or femorotibial PWV (ftPWV), the commonly used PWV index measured outside the main aortic track, reflects mainly the stiffness of the small arteries rather than pure aortic stiffness, its predicted value in cardiovascular disease is still controversial (Boutouyrie et al., 2014; Iulita et al., 2018). Thus, considering the validation in clinic practice, we performed a systematic review and meta-analysis about the association between aortic stiffness measured using the validated aortic PWV and cognitive function, risk of cognitive impairment, or dementia to help clarify the association between aortic stiffness and cognitive function in the aging process.

## Method

This systematic review and meta-analysis was reported, adhering to the Preferred Reporting Items for Systematic Reviews and Meta-analyses (PRISMA) statement and Meta-analysis of Observational Studies in Epidemiology (MOOSE) checklist (Stroup et al., 2000; Liberati et al., 2009).

### Search Strategy and Data Source

We searched for articles published from January 1, 1986, to March 16, 2020, through electronic databases, including PubMed, Cochrane Library, and EMBASE using “aortic stiffness” and “cognitive impairment” as major themes (precise search terms are provided in the Supplementary Material). The search was restricted to articles published in the English language. Also, we reviewed the reference lists of all relevant articles for potentially eligible studies.

### Selection Strategy and Criteria

Two investigators (QL and CHC) independently screened all relevant studies and determined eligibility based on the title, abstract, and full texts. Studies were included if they matched the following inclusion criteria: (1) human studies and full-length publications in peer-reviewed journals; (2) cross-sectional or longitudinal designed studies; (3) in order to comprehensively explore the association between aortic stiffness and cognition in adults, we included the studies with participants aged 18 years or older, regardless of sample size and types of population (including general population or targeting on a particular population with a risk factor or disease); (4) reporting an association between aortic stiffness and cognitive function; (5) evaluating aortic stiffness exclusively using validated PWV measurement along aorta; (6) cognitive function were assessed with validated scales, and mild cognitive impairment (MCI) or dementia was diagnosed based on clinic diagnostic standards or guidelines. Studies were excluded if they met the exclusion criteria: (1) case–control study or placebo-controlled clinical trial (involving a specific intervention); (2) the article did not report an association between aortic stiffness and cognitive impairment; and (3) the aortic stiffness was assessed using PWV measured outside the aortic track, at the upper (baPWV) or lower limb (ftPWV).

### Data Extraction

Two investigators (QL and CHC) independently extracted data from each eligible study. Any disagreements were resolved by consensus or consultation with a third investigator (JHF). The following information was extracted from each eligible study: authors, published year, design (follow-up years for longitudinal studies), country, study population, sample size, male (%), mean age, mean or median aortic PWV value (m/s), cognitive tests, adjusted covariates, and main results. The outcomes for meta-analysis were various domains of cognitive function, cognitive impairment, and dementia. In cognitive function domains, we focused on attention, executive function, global cognitive function, memory, processing speed, and visuospatial ability. For those studies that published more than one article from the same cohort, (1) if they had the same study design and cognitive outcomes, we included only the one with the results either that could be included in meta-analysis or with the largest sample size; (2) if they reported on different cognitive outcomes, we included different cognitive domains' data in each of these articles separately in the analysis. Required metrics not reported in the article were requested from the corresponding authors by email.

### Quality Assessment

Two investigators (QL and JHF) independently assessed the quality of included studies using the modified version of Newcastle Ottawa Scale (NOS) (Wells et al., 2014) (see the Supplementary Material). The NOS includes items on participant selection, the validity of measurements, and whether adjusting associations by systolic and/or mean blood pressure (MBP), age and education, and assessment of outcomes. For cross-sectional studies, the maximum score was 5 points, and scores <3 points were considered as high risk of bias. For longitudinal studies, the maximum score was 8 points, and scores <4 points were considered as high risk of bias.

### Statistical Analysis

All analyses were performed using Comprehensive Meta-Analysis software version 3 (CMA 3.0, Biostat Inc., Englewood, NJ, USA). For cross-sectional studies, Pearson's r correlation coefficients were pooled as the effect size to show the association between aortic PWV and cognitive function (attention, executive function, global cognitive function, memory, processing speed, and visuospatial ability). Multiple scales for one cognitive domain in each study were collapsed into a single effect size (Borenstein and Wiley, 2009). Negative associations indicated that greater stiffness (aortic PWV) was associated with worse cognitive function. Additionally, the r correlation coefficients and 95% CI between aortic PWV and Mini-Mental State Examination (MMSE) scores were synthesized. For longitudinal studies, the odds ratios (ORs) were pooled as effect size to show the association between aortic PWV (the highest stiffness group vs. the lowest group) and risk of cognitive impairment and dementia. Since most studies reported ORs of continuous aortic PWV metric, we also pooled the adjusted ORs per absolute aortic PWV (1 m/s) to explore between constant aortic PWV values and the risks of cognitive impairment or dementia. A random-effects model was used to pool these effect estimates when significant heterogeneity existed among studies.

Among the included studies, a few studies recruited participants specifically with chronic kidney disease (including end-stage renal disease), hypertension, and complain of memory loss. Thus, we divided these studies into three categories according to their participants' condition: chronic kidney disease, hypertension, and complaint of memory loss. Sensitivity analyzes were performed by excluding these three categories of studies one by one from the pooled estimates to show the influence of these specific conditions on the overall effect size and 95% CI. Moreover, we performed subgroup analysis when there were more than three papers in each of the above three categories. Meta-regression analysis was conducted to evaluate the influence of mean age, percentage of male, MBP, and percentage of education of high school or less on the association between aortic stiffness and cognitive decline.

Q test and I-squared statistics were used to examine the heterogeneity across studies, with a *P* ≤ 0.10 and *I*^2^ ≥ 50% indicating significant heterogeneity (Higgins et al., 2003; Borenstein and Wiley, 2009). Egger's test and funnel plot were used to evaluate publication bias, and *P* < 0.05 of Egger's test and/or funnel plot asymmetry was considered as the existence of publication bias (Borenstein and Wiley, 2009).

## Results

### Qualitative Summary Characteristic of Included Studies

**Figure 1:**
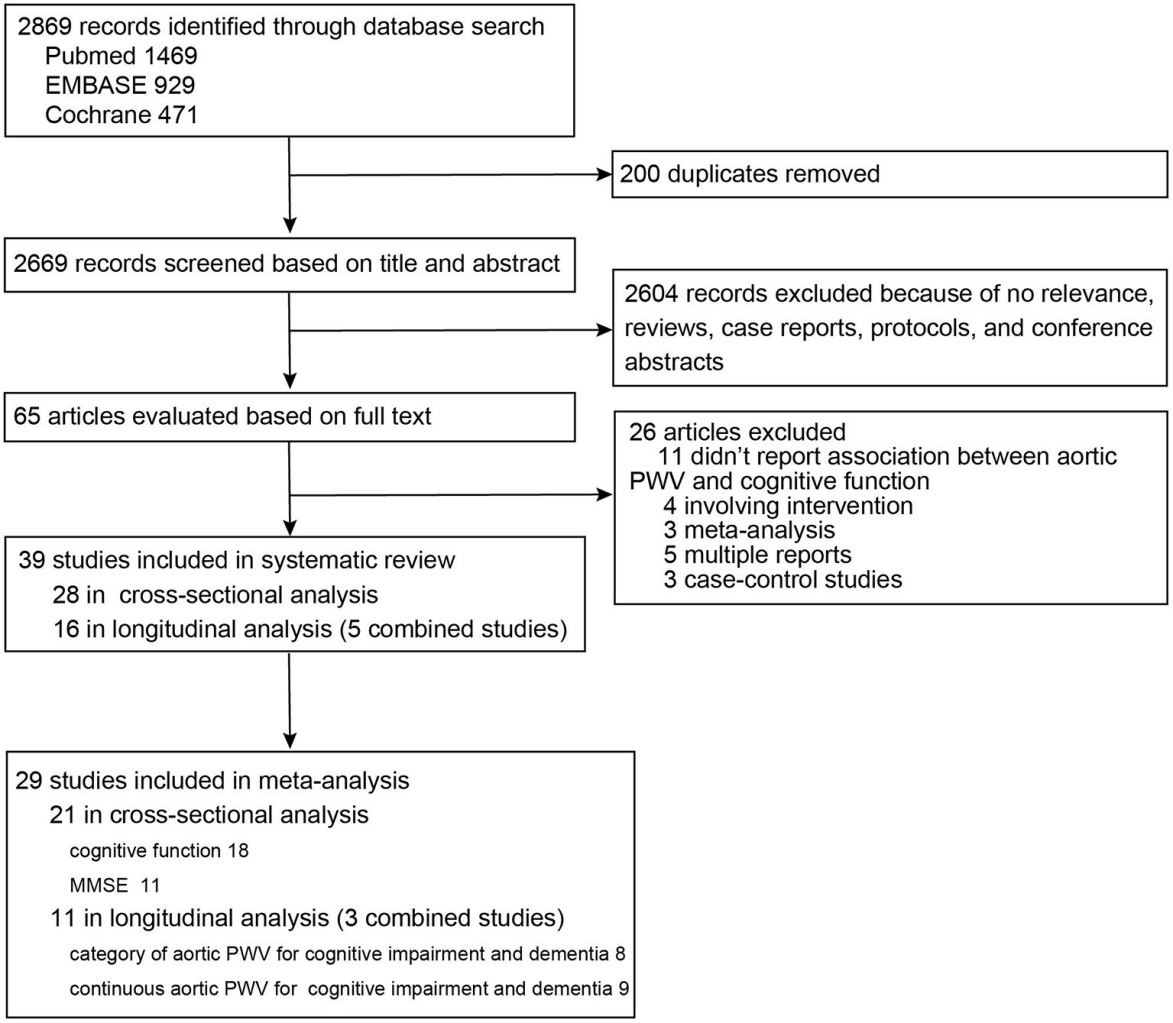
Flow diagram of study selection for systematic review and meta-analysis.

As shown in the diagram of the selection process (Figure 1), among 2,869 records, 65 articles were evaluated based on full text. Finally, 39 studies were summarized for detailed review, including 28 studies with 29,955 participants from 14 different countries in the cross-sectional analysis (Hanon et al., 2005; Scuteri et al., 2005; Poels et al., 2007; Elias et al., 2009; Kearney-Schwartz et al., 2009; Triantafyllidi et al., 2010; Watson et al., 2011; Singer et al., 2013; Nilsson et al., 2014, 2017; Zhong et al., 2014; Cooper et al., 2016; Geijselaers et al., 2016; Lim et al., 2016; Pase et al., 2016b; Riba-Llena et al., 2016; Tasmoc et al., 2016; Kim et al., 2017; Meyer et al., 2017; Suleman et al., 2017; Karasavvidou et al., 2018; Kennedy et al., 2018; Muela et al., 2018; Araghi et al., 2019; DuBose et al., 2019; Palta et al., 2019; Dixon et al., 2020; Zijlstra et al., 2020) and 16 studies with 23,448 participants from seven different countries in the longitudinal analysis (Poels et al., 2007; Scuteri et al., 2007, 2013; Waldstein et al., 2008; Watson et al., 2011; Zeki Al Hazzouri et al., 2013; Watfa et al., 2015; Hajjar et al., 2016; Pase et al., 2016a; Tsao et al., 2016; Kim et al., 2017; Nilsson et al., 2017; Cui et al., 2018; Rouch et al., 2018; Araghi et al., 2019; Menezes et al., 2019) (Supplementary Tables 1, 2). Only the study of Zijlstra et al. measured aortic PWV using MRI and showed median PWV of 9.6 m/s (interquartile range: 7.8–13.0) (Zijlstra et al., 2020). The other studies used cfPWV and showed mean or median PWV ranging from 4.96 to 14.3 m/s. For the cross-sectional study, modified NOS scores ranged from 1 to 5 points, and 10 studies (35.7%) (Scuteri et al., 2005; Kearney-Schwartz et al., 2009; Tasmoc et al., 2016; Kim et al., 2017; Suleman et al., 2017; Karasavvidou et al., 2018; Muela et al., 2018; Araghi et al., 2019; Dixon et al., 2020; Zijlstra et al., 2020) had a low score of 1–2 points. For longitudinal studies, the score ranges from 3 to 8 points, with two studies (12.5%) (Kim et al., 2017; Araghi et al., 2019) having a low score of 3 points (Supplementary Tables 1, 2).

In the cross-sectional studies, some reported a significant association between aortic PWV and cognitive function or cognitive decline (Hanon et al., 2005; Scuteri et al., 2005; Elias et al., 2009; Kearney-Schwartz et al., 2009; Watson et al., 2011; Nilsson et al., 2014; Zhong et al., 2014; Cooper et al., 2016; Lim et al., 2016; Pase et al., 2016b; Tasmoc et al., 2016; Meyer et al., 2017; Kennedy et al., 2018; Muela et al., 2018; Araghi et al., 2019; DuBose et al., 2019; Palta et al., 2019; Dixon et al., 2020; Zijlstra et al., 2020), while some did not support significant association between aortic PWV and cognitive function or dementia (Poels et al., 2007; Triantafyllidi et al., 2010; Singer et al., 2013; Geijselaers et al., 2016; Riba-Llena et al., 2016; Kim et al., 2017; Nilsson et al., 2017; Suleman et al., 2017) (Supplementary Table 1). Among them, studies that included exclusive participants with chronic kidney disease (Tasmoc et al., 2016; Kim et al., 2017; Karasavvidou et al., 2018; Zijlstra et al., 2020) or hypertension (Triantafyllidi et al., 2010; Riba-Llena et al., 2016; Muela et al., 2018) mainly did not support the association between aortic stiffness and cognitive decline. However, those studies with participants complaining of memory loss showed a significant association (Hanon et al., 2005; Scuteri et al., 2005; Kearney-Schwartz et al., 2009; Dixon et al., 2020) (Supplementary Table 1).

In longitudinal studies, 13 studies with 17,727 participants (followed up 1–15 years) suggested significant associations between aortic PWV and cognitive decline or dementia (Scuteri et al., 2007, 2013; Waldstein et al., 2008; Watson et al., 2011; Zeki Al Hazzouri et al., 2013; Watfa et al., 2015; Hajjar et al., 2016; Pase et al., 2016a; Tsao et al., 2016; Cui et al., 2018; Rouch et al., 2018; Araghi et al., 2019; Menezes et al., 2019), in which three studies included participants with complaints of memory loss (Scuteri et al., 2007, 2013; Rouch et al., 2018), whereas two studies with 5,721 participants (followed up about 4 years) (Poels et al., 2007; Nilsson et al., 2017) and one study with 135 hemodialysis participants (followed up 1 year) (Kim et al., 2017) did not find association between aortic PWV and cognitive impairment (Supplementary Table 2).

### Aortic Pulse Wave Velocity and Cognitive Function

**Figure 2:**
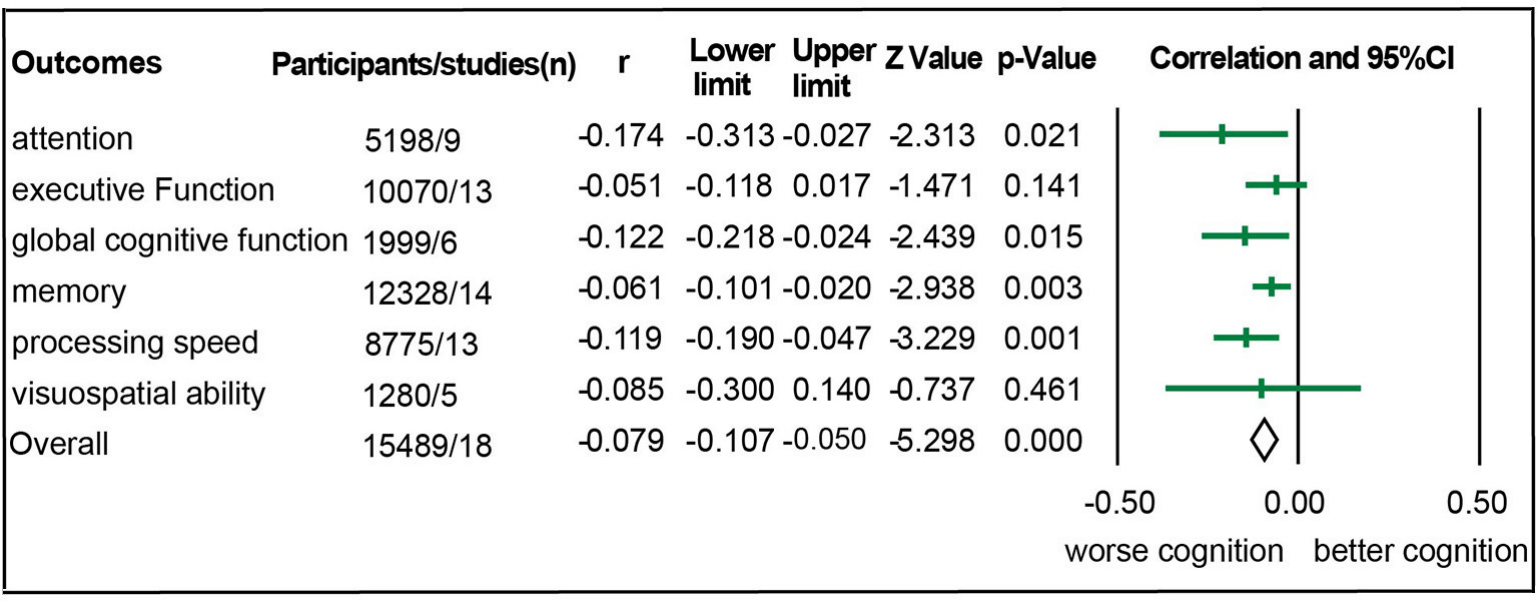
Forest plot for association between aortic stiffness and domains of cognitive function.

Eighteen studies with 15,489 participants were eligible for meta-analysis of association between aortic PWV and cognitive function (Hanon et al., 2005; Poels et al., 2007; Elias et al., 2009; Watson et al., 2011; Singer et al., 2013; Nilsson et al., 2014; Zhong et al., 2014; Cooper et al., 2016; Geijselaers et al., 2016; Lim et al., 2016; Pase et al., 2016b; Riba-Llena et al., 2016; Tasmoc et al., 2016; Kennedy et al., 2018; Muela et al., 2018; DuBose et al., 2019; Dixon et al., 2020; Zijlstra et al., 2020). Among them, there were 157 chronic kidney disease participants (Tasmoc et al., 2016; Zijlstra et al., 2020), 976 hypertension participants (Riba-Llena et al., 2016; Muela et al., 2018), and 364 participants complaining of memory loss (Hanon et al., 2005; Dixon et al., 2020). As shown in Figure 2, we detected a significant association between aortic PWV and attention (*r* = −0.174), global cognitive function (*r* = −0.122), memory (*r* = −0.061), and processing speed (*r* = −0.119) in all participants (Figure 2, Table 1). Nevertheless, significant heterogeneity existed among studies (Supplementary Table 3). After studies with participants of chronic kidney disease, of hypertension, and complaining loss of memory were excluded, the aortic PWV was still statistically associated with memory (*r* = −0.022) and processing speed (*r* = −0.048) (Table 1), without significant heterogeneity and publication bias (Supplementary Table 3).

**Table 1:** Sensitive analyses of association between aortic PWV and cognitive function.

**Outcomes**	**Analysis 1**			**Analysis 2**			**Analysis 3**			**Analysis 4**		
	**Studies**	**Participants**	***r* (95% CI)**	**Studies**	**Participants**	***r* (95% CI)**	**Studies**	**Participants**	***r* (95% CI)**	**Studies**	**Participants**	***r* (95% CI)**
Attention	9	5,198	−0.174 (−0.313, −0.027)^*^	7	5,041	0.006 (−0.051, 0.063)	5	4,335	0.031 (−0.009, 0.071)	4	4,279	0.034 (−0.012, 0.079)
Executive Function	13	10,070	−0.051 (−0.118, 0.017)	11	9,913	−0.006 (−0.061, 0.050)	9	9,207	0.017 (−0.043, 0.076)	8	9,151	0.003 (−0.054, 0.059)
Global cognitive function	6	1,999	−0.122 (−0.218, −0.024)^*^	6	1,999	−0.122 (−0.218, −0.024)^*^	4	1,293	−0.113 (−0.255, 0.034)	3	1,127	−0.055 (−0.143, 0.033)
Memory	14	12,328	−0.061 (−0.101, −0.020)^*^	12	12,171	−0.034 (−0.063, −0.004)^*^	10	11,465	−0.022 (−0.041, −0.003)^*^	9	11,409	−0.022 (−0.042, −0.001)^*^
Processing speed	13	8,775	−0.119 (−0.190, −0.047)^*^	11	8,618	−0.056 (−0.094, −0.017)^*^	10	8,476	−0.048 (−0.081, −0.016)^*^	9	8,420	−0.048 (−0.082, −0.014)^*^
Visuospatial ability	5	1,280	−0.085 (−0.300, 0.140)	5	1,280	−0.085 (−0.300, 0.140)	4	1,138	−0.073 (−0.328, 0.192)	4	1,138	−0.073 (−0.328, 0.192)

*Analysis 1 included all the eligible studies; Analysis 2 excluded studies with participant of chronic kidney disease based on analysis 1; Analysis 3 further excluded studies with participants of hypertension based on analysis 2; Analysis 4 further excluded studies with participants complaining loss of memory based on analysis 3 and just included studies with participants from general older adults*.

* *P < 0.05*.

A significant association between aortic PWV and MMSE scores was detected among 11 studies with 9,034 participants (*r* = −0.11, 95% CI −0.15 to −0.07) (Hanon et al., 2005; Scuteri et al., 2005; Poels et al., 2007; Triantafyllidi et al., 2010; Nilsson et al., 2014; Zhong et al., 2014; Lim et al., 2016; Tasmoc et al., 2016; Karasavvidou et al., 2018; Muela et al., 2018; Dixon et al., 2020), but there were significant heterogeneity and publication bias (Supplementary Table 4, Supplementary Figure 5). After studies with participants with specific disease or condition (Hanon et al., 2005; Scuteri et al., 2005; Triantafyllidi et al., 2010; Tasmoc et al., 2016; Karasavvidou et al., 2018; Muela et al., 2018; Dixon et al., 2020) were excluded, the significance disappeared, while in the subgroup of participants complaining of memory loss (Hanon et al., 2005; Scuteri et al., 2005; Dixon et al., 2020), the aortic PWV was significantly associated with MMSE score (*r* = −0.27), and there were no heterogeneity and significant publication bias (Supplementary Table 4). These results supported aortic stiffness associated with cognitive impairment. However, as a screening test for dementia, the MMSE scale might not be a validated and sensitive tool for detecting cognitive impairment in the general population (Waldstein et al., 2008; Pase et al., 2012).

### Aortic Pulse Wave Velocity and Cognitive Impairment or Dementia

**Figure 3:**
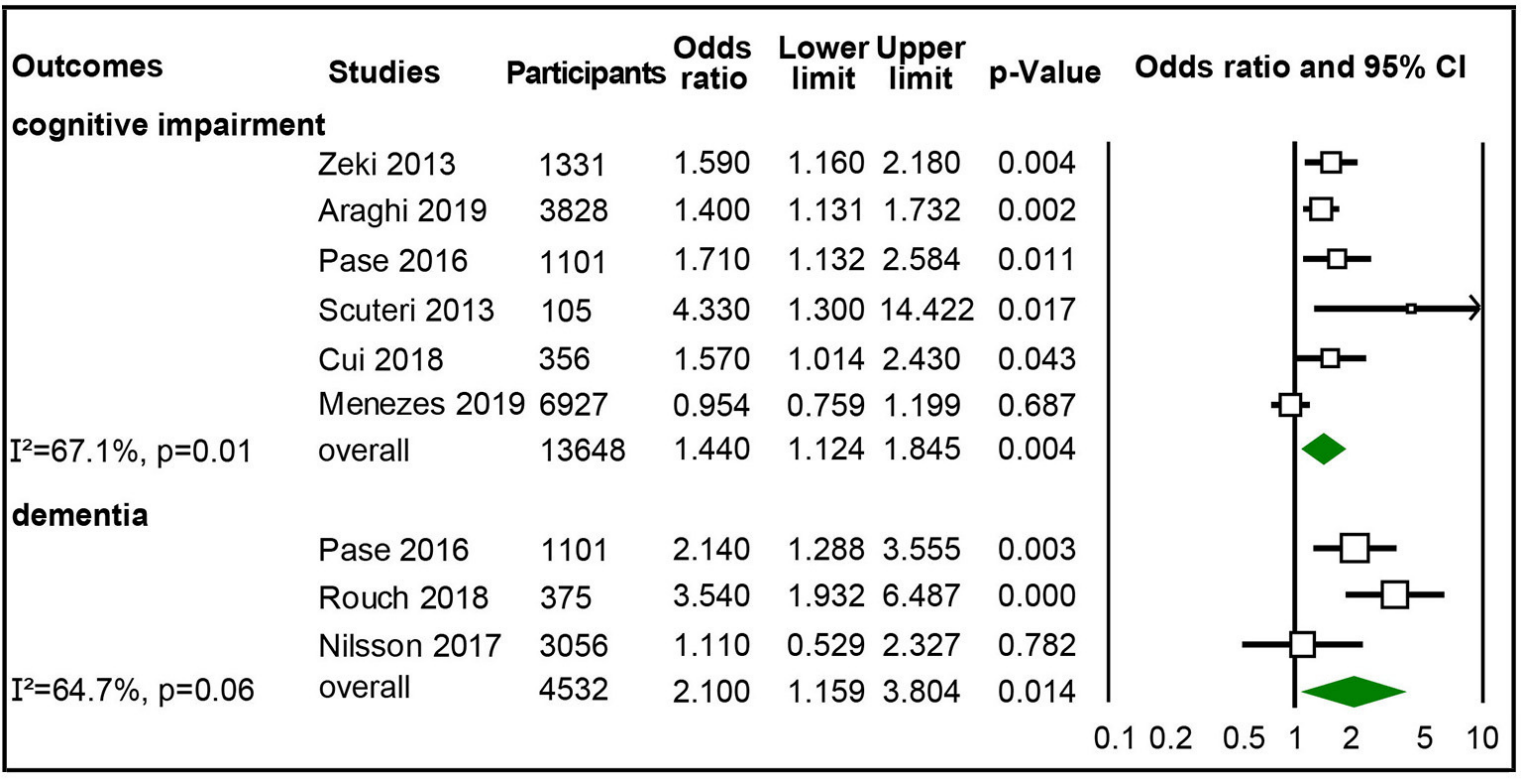
Forest plot of association between categorical aortic pulse wave velocity (PWV) (high vs. low) and cognitive impairment and dementia.

Six studies with 13,648 participants were included in synthesizing adjusted ORs of the highest vs. lowest category of aortic PWV to cognitive impairment (Scuteri et al., 2013; Zeki Al Hazzouri et al., 2013; Pase et al., 2016a; Cui et al., 2018; Araghi et al., 2019; Menezes et al., 2019), and three studies with 4,532 participants were synthesized for adjusted ORs for dementia (Pase et al., 2016a; Nilsson et al., 2017; Rouch et al., 2018). The pooled results showed that the highest category of aortic PWV independently increased risk of cognitive impairment (OR 1.44, 95% CI 1.124–1.845) and dementia (OR 2.1; 95% CI 1.159–3.804) than the lowest group of aortic PWV, though with moderate heterogeneity (Figure 3), but no significant publication bias (Supplementary Figure 6). After studies with participants complaining of memory loss (Scuteri et al., 2013; Rouch et al., 2018) were excluded, the significantly increased risk for cognitive impairment remained, but not for dementia (Supplementary Figure 2).

**Figure 4:**
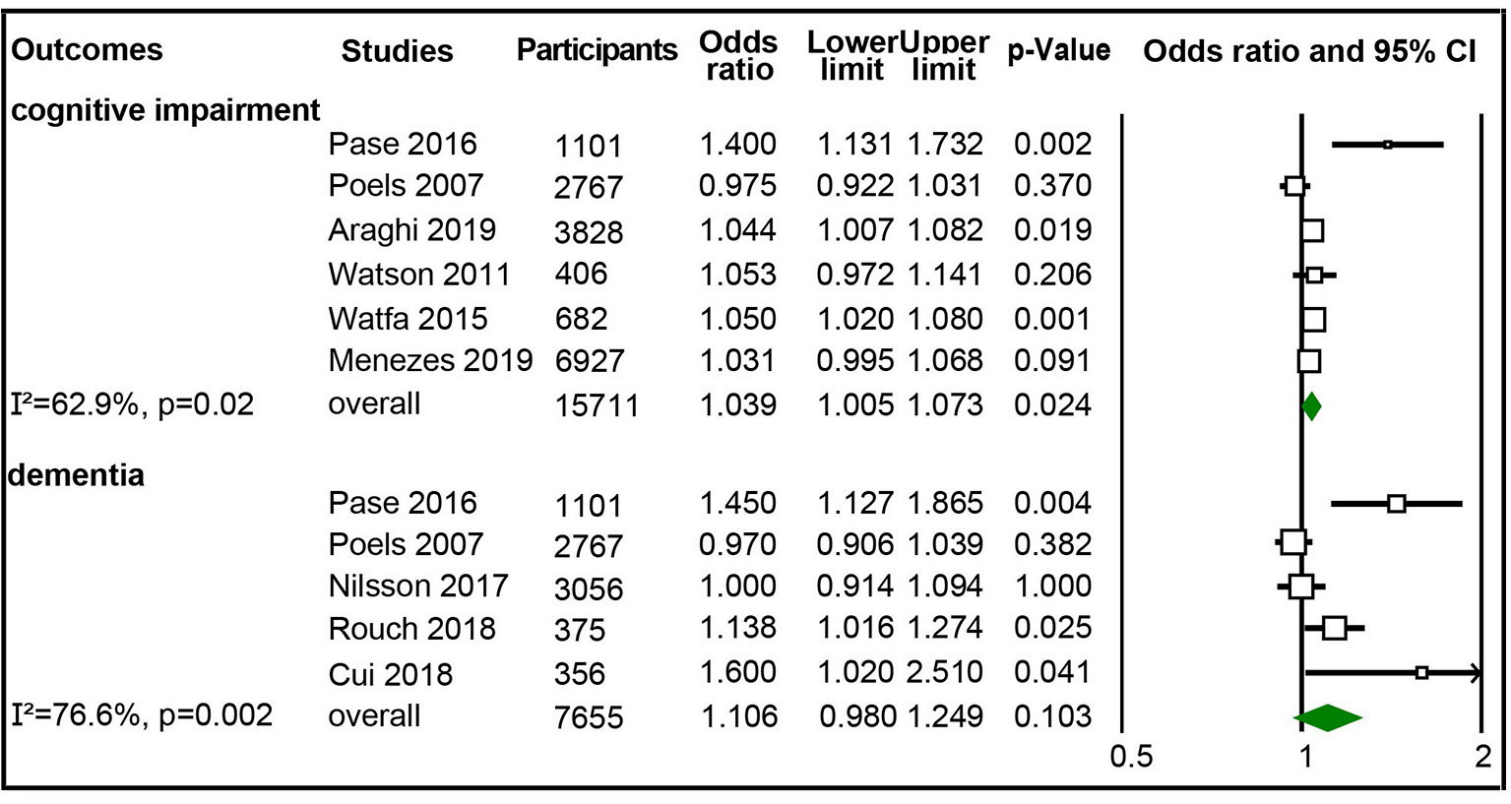
Forest plot of association between continuous aortic pulse wave velocity (PWV) (m/s) and cognitive impairment and dementia.

We also calculated the pooled ORs of continuous aortic PWV (m/s) to cognitive impairment or dementia. It showed that the cognitive impairment risk increased 3.9% (OR 1.039; 95% CI 1.005–1.073) per 1 m/s of aortic PWV increase from six studies (15,711 participants) reporting risk of cognitive impairment (Poels et al., 2007; Watson et al., 2011; Watfa et al., 2015; Pase et al., 2016a; Araghi et al., 2019; Menezes et al., 2019). There were moderate heterogeneity and no publication bias (Figure 4, Supplementary Figure 6). However, for the five studies with 7,655 participants reporting risk for dementia (Poels et al., 2007; Pase et al., 2016a; Nilsson et al., 2017; Cui et al., 2018; Rouch et al., 2018), we did not detect a significant association between continuous aortic PWV (m/s) and the risk of dementia (Figure 4, Supplementary Figure 3).

### Meta-Regression Analysis

The meta-regression analysis showed that age, the proportion of males, and MBP significantly increased the association between aortic PWV and memory or processing speed in all participants (Supplementary Table 5). However, the significance disappeared after excluding studies investigating this association under specific diseases or conditions (Supplementary Table 6). Besides, we found that age significantly increased the risk of cognitive impairment of high vs. low aortic PWV in all participants (Supplementary Table 5, Supplementary Figure 4). The influence of age on this association remained after excluding participants with specific diseases or conditions (Supplementary Tables 5, 6). However, neither age nor other listed variables had an impact of the association between continuous aortic PWV (m/s) and cognitive impairment risk (Supplementary Tables 5, 6).

## Discussion

This comprehensive systematic review and meta-analysis showed that aortic stiffness measured with aortic PWV was inversely associated with the function of memory and processing speed, and aortic PWV was an independent predictor for cognitive impairment. Besides, age could increase the association between high aortic PWV and the risk of cognitive impairment.

The association between aortic stiffness and memory was thought to be mainly due to the microvascular injury in deep white matter (Mitchell et al., 2011; Kloppenborg et al., 2014; Cooper et al., 2016), and the statistical mediation analysis showed that cerebrovascular resistance (52% of indirect effect) and white matter hyperintensities (41% of indirect effect) accounted for major observed relation between cfPWV and memory (Cooper et al., 2016). Besides, the excessive pulsatile damage from aortic stiffness to the medial temporal lobe and hippocampus may contribute to poor memory as well (Wåhlin et al., 2014; Lilamand et al., 2016). It was reported that the major brain structural changes caused by aortic stiffness was white matter lesion (Tarumi et al., 2015; van Sloten et al., 2015), which was thought to preferentially cause the decline of processing speed and executive function (Kloppenborg et al., 2014; Biesbroek et al., 2017). However, we just detected a significant correlation between aortic stiffness and processing speed but not executive function. The non-significant association between aortic stiffness and executive function in our analysis may due to the heterogeneity among studies or the non-linear association (Nilsson et al., 2014; Zhong et al., 2014; Dixon et al., 2020), which prevented the detection of significant linear association using the correlation coefficient as the effect size. Several pathophysiologic mechanisms might be involved in the diminishing effect of aortic stiffness on cognitive function. Besides the microvascular damage caused by excessive pulsatile load (Mitchell et al., 2011; van Sloten et al., 2015; Lilamand et al., 2016), the reduced cerebral perfusion due to aortic stiffness may aggravate the white matter lesion and brain atrophy (Tarumi et al., 2011). Moreover, the Aβ deposition was found to play an important role in the association between aortic stiffness and cognitive impairment (Hughes et al., 2013, 2018).

Besides increased risk for cognitive impairment, our results indicated that the high aortic stiffness increased the risk of dementia by 2-fold. But the significant association disappeared after excluding the study of Rouch et al. We speculated that this may due to the limited studies for synthesized analysis and short follow-up years. Although the study by Rouch et al. just followed up in a relatively short period of 4.5 years, including specific participants of MCI in their study would make it more sensitive to detect the independent association between aortic stiffness and risk of dementia (Rouch et al., 2018).

Additionally, the meta-regression analysis indicated that age increased the risk of cognitive impairment caused by high aortic PWV. This is consistent with studies that showed that the interaction of PWV and age (PWV × age) increased the magnitude of associations between PWV and cognitive performance (Elias et al., 2009; Pase et al., 2016b; Menezes et al., 2019). Since both aortic stiffness and cognitive impairment are age-related changes (Langa and Levine, 2014; Iulita et al., 2018), there should be vicious loop between age, aortic stiffening, and cognitive decline. Thus, it is important to make early interventions to prevent progression of aortic stiffness to delay cognitive impairment and dementia. A recently published meta-analysis using Cohen's d index as effect sizes also showed a negative relationship between arterial stiffness with executive function and memory, but they did not find age to modify the strength of this association (Alvarez-Bueno et al., 2020). This might because they included studies with PWV index involving the stiffness of the peripheral arteries (i.e., baPWV), which could diminish the direct effect of age on aortic stiffness. Besides, they did not analyze the association between PWV and risk for cognitive impairment, a cumulative result of cognitive function decline, in which the effect of age on this association should be more pronounced.

A few limitations should be noticed. First, the limited amount of studies included in the meta-analysis may prevent the full interpretation of the results. But our results supported that the high aortic stiffness at least associated with memory and processing speed decline and increased cognitive impairment risk. Second, the variables for adjustment varied from study to study, which increased the heterogeneity of studies as well as the variation of true effect size. And it is possible that residual confounding remains in some studies, which may prevent detecting the statistical significance for some domains of function that would have had a significant association with aortic stiffness. But this should have less influence on the significance of association that we have already found in this meta-analysis. Finally, we did not pool the correlation of aortic PWV and domains of cognitive function in longitudinal studies due to the limited available data. Since there were studies that showed a faster decline in several domains of cognitive function with higher aortic stiffness (Hajjar et al., 2016; Menezes et al., 2019), it would be more convincing if we further confirmed this longitudinal association in our quantitative analysis.

## Conclusion

In summary, this systematic review and meta-analysis suggested that aortic stiffness is inversely associated with cognitive function, an independent predictor for cognitive impairment, and a potential risk factor for dementia, especially in the elderly. This study supports the assessment of the aortic PWV in routine clinical practice for primary prevention to slow down early the progression of cognitive decline.

## Data Availability Statement

The original contributions presented in the study are included in the article/Supplementary Material, further inquiries can be directed to the corresponding author/s.

## Author Contributions

QL and JF: study conception and design. NC and LH: supervision and administration. QL, JF, MM, and NC: writing-manuscript preparation and intellectual input. QL, JF, CC, NC, and LH: data interpretation. QL, JF, CC, and SD: data analysis. QL, JF, CC, SD, LG, JB, and YL: experiment or data collection. All authors contributed to the article and approved the submitted version.

## Conflict of Interest

The authors declare that the research was conducted in the absence of any commercial or financial relationships that could be construed as a potential conflict of interest.
