# Supplementary material for: Association of Aortic Stiffness and Cognitive Decline: A Systematic Review and Meta-Analysis
Source: Front Aging Neurosci. 2021 Jun 24;13:680205. doi: 10.3389/fnagi.2021.680205 (PMC8261283; doi:10.3389/fnagi.2021.680205)
Supplement: Supplementary file 1 [file Data_Sheet_1.docx]

**Literature search**

All searches were setting limitation for 1) publication date from 1986/01/01 (except for Cochran library that can’t define specific publication date); 2) language of English; and 3) human study. Search terms are provided below: Search terms and strategies for each database are provided below:

*Aortic stiffness:* Aortic stiffness OR Stiffness, Vascular OR Vascular Stiffnesses OR Arterial Stiffness* OR Stiffness, Arterial OR Aortic Stiffness* OR Stiffness, Aortic OR Carotid Femoral Pulse Wave Velocity OR cf-PWV OR Carotid-Femoral Pulse Wave Velocities OR Carotid Femoral Pulse Wave Velocities OR pulse pressure*

*Cognitive impairment*: ((Cogniti* OR neurocognit* OR neuropsychologic* OR mental* OR memory OR executive function) AND (impairment* OR dysfunction* OR disorder* OR decline OR deterioration* OR defect*)) OR dementia OR amentia OR Alzheimer*

**Newcastle-Ottawa quality assessment scale**

For cross-sectional studies, the item 2 and 4 of selection and item 2 and 3 of outcome in original Newcastle Ottawa Scale (NOS) for cohort studies were not counted for their nature of cross-sectional design.

For longitudinal studies, items 2 of selection in original Newcastle Ottawa Scale (NOS) were not counted since the total cohort was under “exposed” to the risk factor of arterial stiffness.

And a study can be awarded a maximum of one star for each numbered item within the selection and outcome categories. A maximum of two stars can be given for comparability.

In general, the modified Newcastle Ottawa Scale (NOS) for this study are listed below:

- *Selection*

1) Representativeness of the cohort

a) truly representative of the average population in community*****

b) somewhat representative of the average population in community (eg. Middle age or older general population) *****

c) selected group of users e.g. nurses, volunteers

d) no description of the derivation of the cohort

2) Ascertainment of aortic stiffness

a) use of an approved device and measurement for aortic PWV *****

b) not an approved device

c) written self-report

c) no description

3) Demonstration that outcome of interest was not present at start of study (at start of the study abnormal performance on cognitive function tests were excluded)

a) yes *****

b) no

- *Comparability*

1) Comparability of cohorts on the basis of the design or analysis

a) study controls for blood pressure (systolic, diastolic and (or) mean blood pressure) *****

b) study controls for age and education *****

- *Outcome*

1) Assessment of outcome

a) independent blind assessment *****

b) record linkage (for dementia only) *****

c) self-report

d) no description

2) Was follow-up long enough for outcomes to occur

a) yes (median/mean follow-up duration > 5 year) *****

b) no

3) Adequacy of follow-up of cohorts

a) complete follow-up - all subjects accounted for *****

b) subjects lost to follow-up unlikely to introduce bias - small number lost - >80 % follow-up, or description provided of those lost *****

c) follow up rate <80% and no description of those lost to follow-up

d) no statement

**
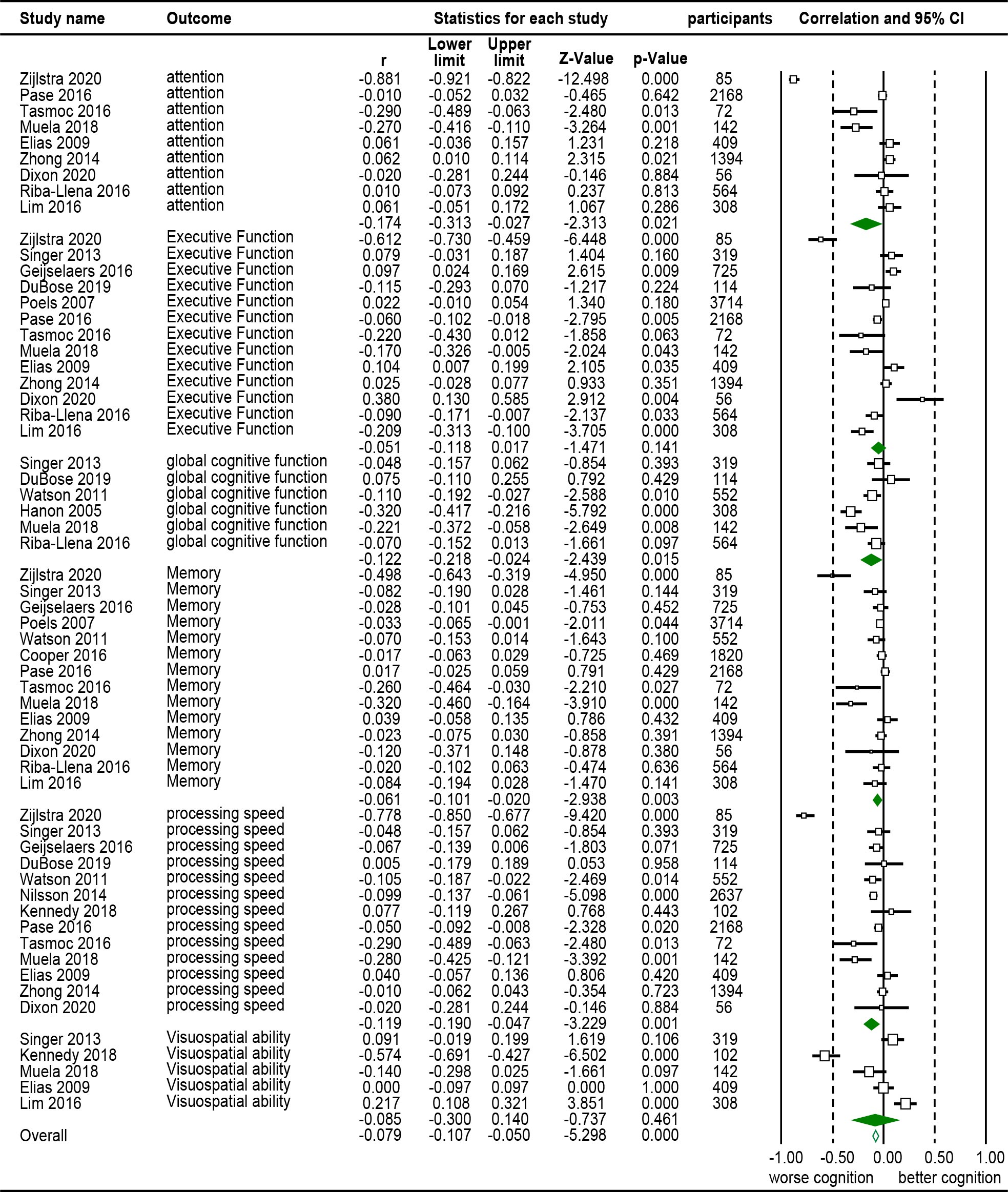
**

**Figure S1.** Cross-sectional Forest Plot of aortic PWV and different domains of cognitive function.


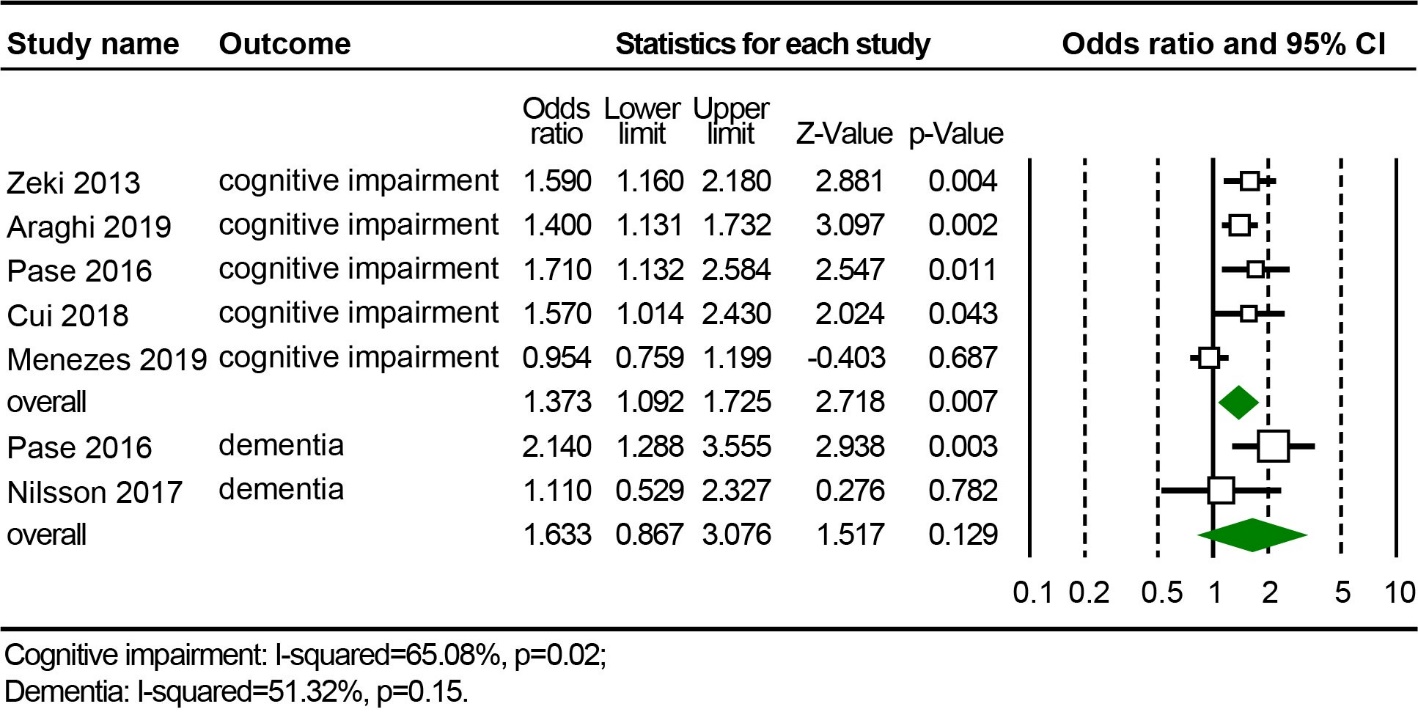


**Figure S2.** Longitudinal Forest Plot of association between aortic PWV (high vs. low) and cognitive impairment and (or) dementia after excluding participants with special disease.


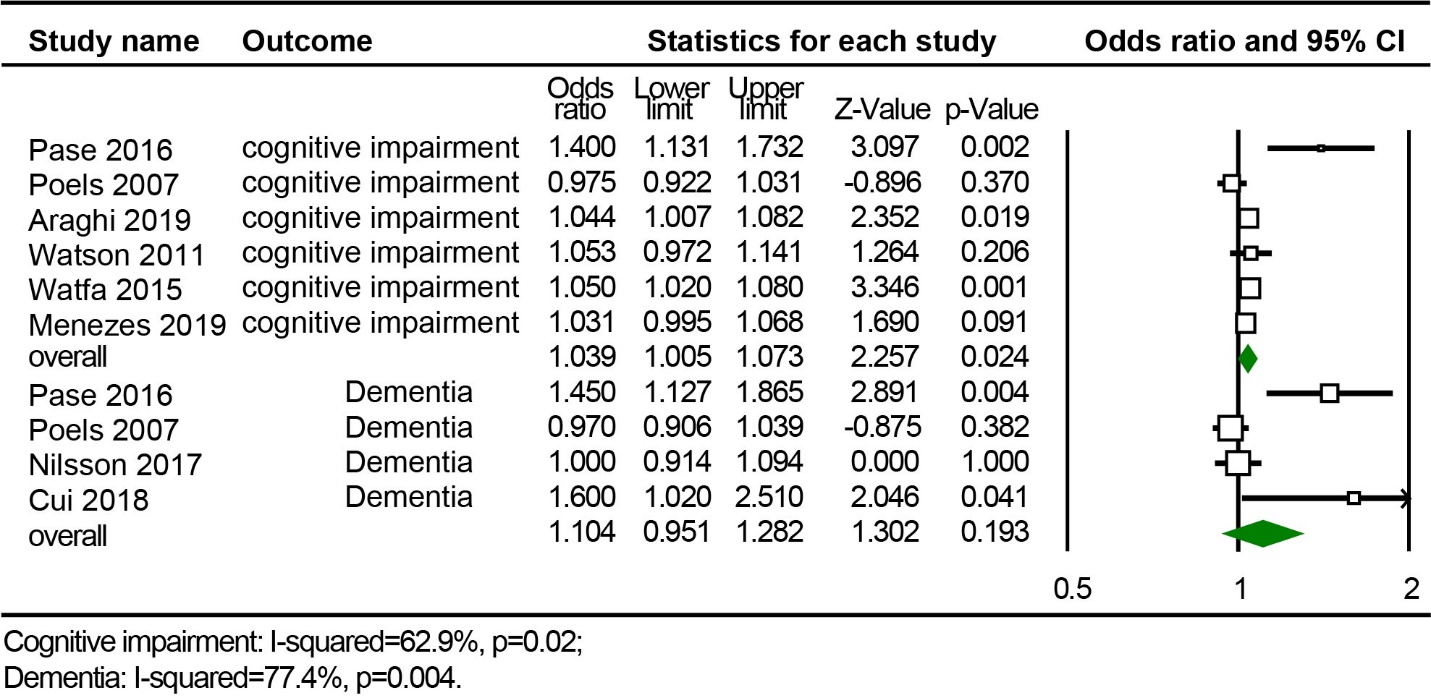


**Figure S3.** Longitudinal Forest Plot of association between continuous aortic PWV (m/s) and cognitive impairment and (or) dementia after excluding participants with special disease.

**
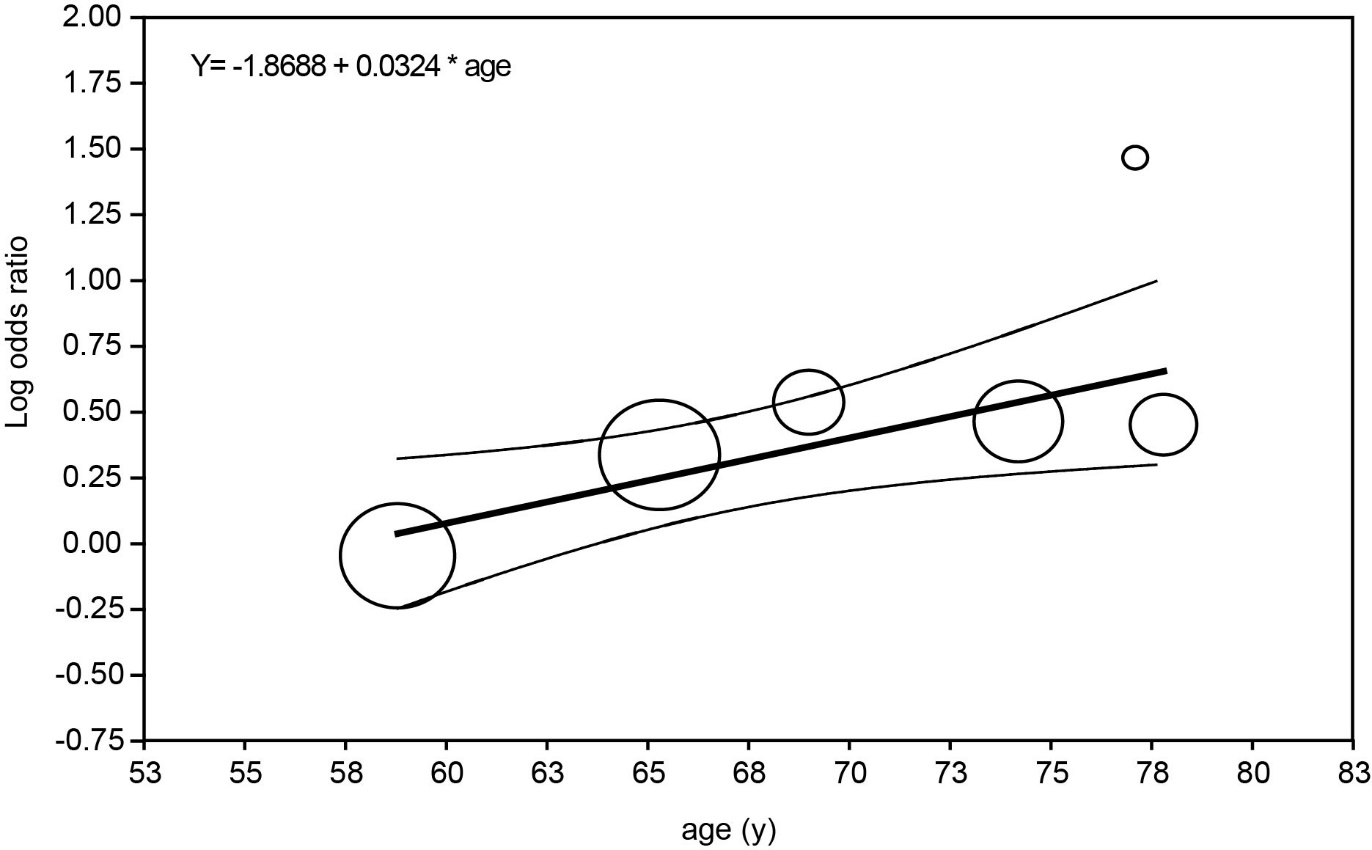
**

**Figure S4.** Bubble plot with a fitted meta-regression line and 95% confidence interval for association between age and log (OR) value of aortic stiffness on cognitive impairment for all the included studies (random-effects model). Circle size is proportional to that study's weight in the analysis.


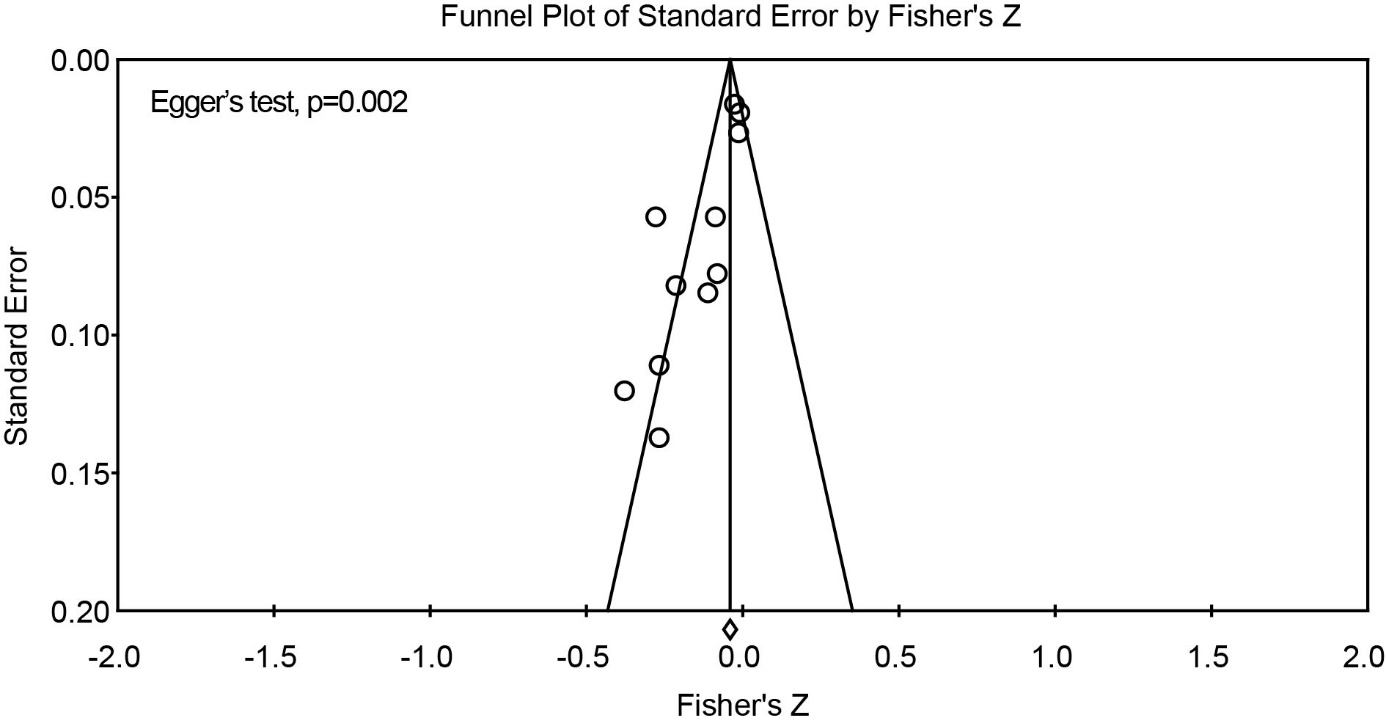


**Figure S5.** Funnel plot of cross sectional analysis association between aortic PWV and MMSE score.


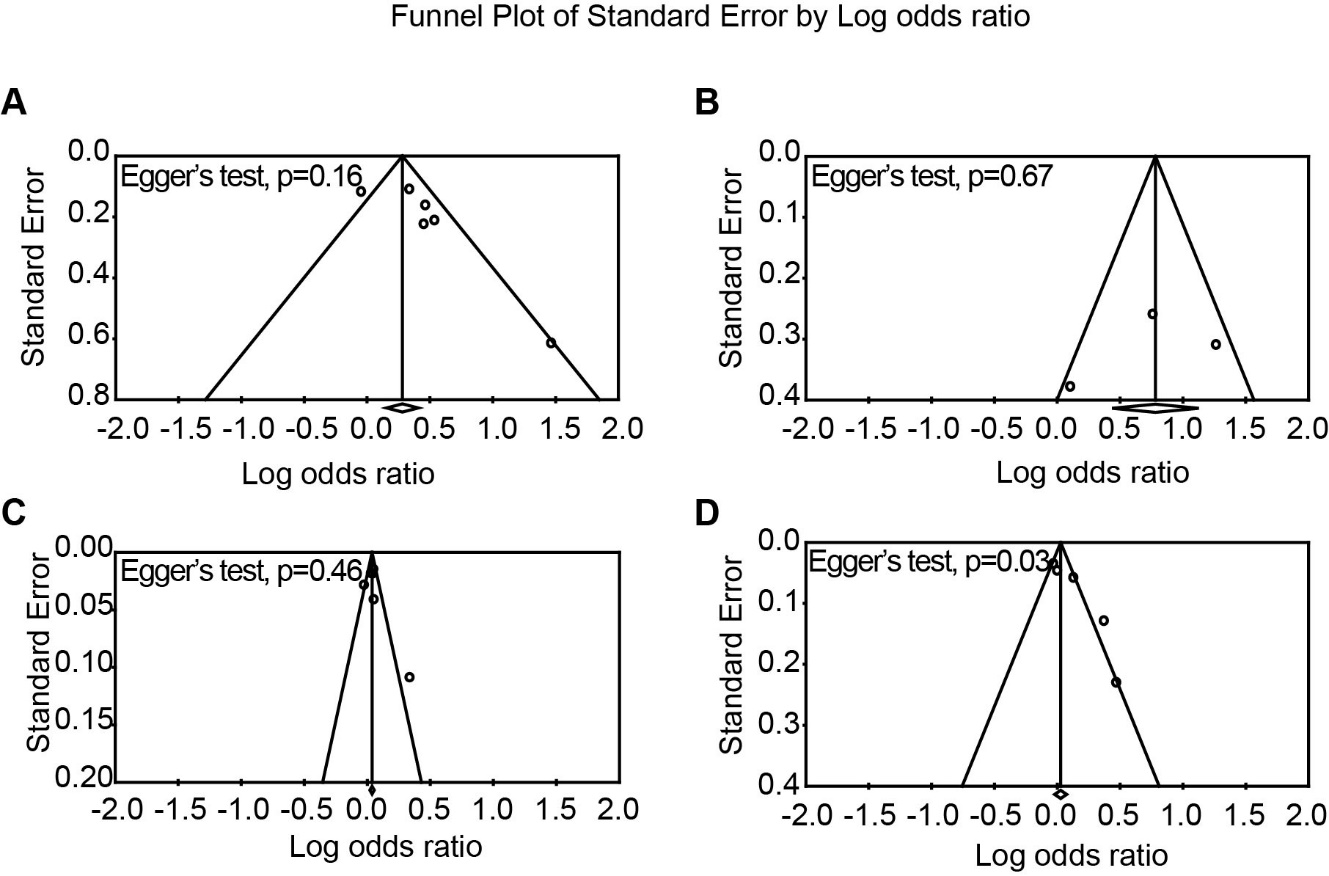


**Figure S6.** Funnel plot of longitudinal analysis association between high versus low aortic PWV and cognitive impairment (A) and dementia (B). And funnel plot of longitudinal analysis association between continuous aortic PWV (m/s) and cognitive impairment (C) and dementia (D).
