# Supplementary material for: Association of Aortic Stiffness and Cognitive Decline: A Systematic Review and Meta-Analysis
Source: Front Aging Neurosci. 2021 Jun 24;13:680205. doi: 10.3389/fnagi.2021.680205 (PMC8261283; doi:10.3389/fnagi.2021.680205)
Supplement: Supplementary file 2 [file Table_1.docx]

**Table S1 characteristic of cross-sectional studies**

| **First author years** | **country** | **study population** | **sample size** | **age**^†^ **years** | **Male %** | **aortic PWV** ^‡^ **m/s** | **outcomes and cognition test** | **results** | **adjusted variables** | **m NOS** |
| --- | --- | --- | --- | --- | --- | --- | --- | --- | --- | --- |
| Singer 2013 (Singer et al., 2013) | Australia | non-demented community dwelling adults aged 70–90 years from MAS Study | 319 | 79.6 (4.2) | 47% | 11.2 (2.4) | **processing speed:** Digit Symbol Coding and Trail Making Test A; **Memory:** Logical Memory Story A (delayed), Rey Auditory Visual Verbal Learning Test (RAVLT) (total learning; trials 1–5, short-term recall; trial 6 and long-term recall; trail 7) and the Benton Visual Retention Test (BVRT); **Language:** Animal Naming and the 30-item Boston Naming Test; **Executive Function:** Phonemic Fluency (FAS), Trail Making Test B and the Stroop Test; **Visuospatial ability:** Block Design. composite **global cognition function** was created by compromising the domains mentioned. | There was no association between cfPWV and cognition after Bonferroni correction for multiple testing | sex, age and years of education; systolic blood pressure, pulse rate, depression, apolipoprotein E, Body Mass Index, stroke, transient ischemic attack, myocardial infarction, angina, smoking, alcohol, diabetes mellitus, cholesterol, anti-hypertensive drugs, and history of hormone replacement therapy. | 5 |
| Zijlstra 2020 (Zijlstra et al., 2020) | Netherlands | patients ≥65 years old reaching ESRD from COPE study | 85 | 75.6 (6.9) | 66% | 9.6 (7.8-13.0) | **Memory:** 15-Word Verbal Learning Test (15-WVLT), Visual Association Test (VAT); **Executive functioning:** Trail Making Test B (TMTB), Stroop Color and Word Test (SCWT) card III (interference card); **Psychomotor speed** : Letter Digit Substitution Test (LDST), Trail Making Test A (TMTA) and SCWT card II (naming colored patches). | Although there were clinically relevant associations of high PWV with poor cognition in all domains, after adjustment for age, sex and education only the Trail Making Test A remained statistically significant (p=0.030) | age, sex and education | 1 |
| Araghi 2019 (Araghi et al., 2019) | British | London-based British civil servants | 3828 | 65.1 ( 5.2) | 74.80% | middle third: 7.41–8.91 | **Memory:** 20-word list; Reasoning:AH4-I (Alice Heim 4-I); **Verbal Fluency:** “S” words and “animal” word; | A cross-sectional association was seen for the highest third cfPWV with all cognitive domains (P < 0.05), compared to the lowest third. | age, sex, ethnicity and employment grade | 2 |
| Watson 2011 (Watson et al., 2011) | USA | well-functioning older adults from Health ABC study | 552 | 73.1 (2.7) | 48% | 8.86 (3.89) | **global cognitive function:** Modified Mini-Mental Status Exam (3MS); **verbal learning and memory:** Buschke Selective Reminding Test (SRT); **psychomotor speed:** Boxes and Digit Copying (BDC) tests; **perceptual speed:** Pattern and Letter Comparison (PLC) tests. | After adjustment for demographics, vascular risk factors, and chronic conditions, each 1 SD higher cfPWV (389 cm/s) was associated with poorer cognitive function: −0.11 SD for global function (SE = 0.04, p < .01), −0.09 SD for psychomotor speed (SE = 0.04, p = .03), and −0.12 SD for perceptual speed (SE = 0.04, p < .01). | age, sex, race, education, and clinic site; body mass index, mean arterial pressure, cholesterol, heart rate, smoking status, physical activity, depressive symptoms, prevalent hypertension, coronary heart disease, cerebrovascular disease, and diabetes mellitus | 5 |
| Pase 2016 (PaseHimali et al., 2016) | USA | young to middle age adults from Framingham Heart Study | 3207 | 46 (9) | 47% | 6.8 (6.1–7.7) | **processing speed and executive function:** Trail Making A and B, the Victoria Stroop interference task; **verbal memory:** Visual Reproductions delayed; **visuoperceptual skills:** the Hooper Visual Organization Test; **working memory:** digit span forward and backward; | higher aortic stiffness was associated with poorer processing speed and executive function (Trail Making B–A; β±SE, −0.08±0.03; P<0.01) | age, sex, and time to neuropsychological testing or MRI; prevalent diabetes mellitus, atrial fibrillation, current smoking, prevalent cardiovascular disease, total cholesterol, high-density lipoprotein cholesterol, depressive symptoms, waist to hip ratio, and treatment for hypertension and mean arterial pressure. | 4 |
| Triantafyllidi 2010 (Triantafyllidi et al., 2010) | Greece | recently diagnosed and never-treated stage I–II essential hypertension | 168 | 53 (12) | 53% | 10.4(2) | **MMSE score** | cfPWV was related with MMSE(r=0.34), but in Multiple regression analysis no significant relationship. | Age, sex, body mass index, atherosclerotic factors (clinic or 24-h BP, smoking, dyslipidemia) | 3 |
| Tasmoc 2016 (Tasmoc et al., 2016) | Romania | hemodynamic stable patients on hemodialysis treatment | 72 | 56.5 (13.9) | 44.4% | 8.98(2.97) | **MMSE score; the MiniCog test; Trail Making Test A and B (TMTA, TMTB)** | cfPWV had a significant moderate negative relationship with the MMSE score (r = −0.36, P < 0.01) and with the MiniCog score (r = −0.26, P = 0.02) and a significant moderate association with the number of seconds required for TMTA completion (r = 0.29, P = 0.01), but not with TMTB (s); | age and gender; dialysis vintage, associated cardiovascular conditions (hypertension, heart failure, coronary artery disease, permanent rhythm disorders, peripheral arterial disease) and associated neurological conditions (hemorrhagic/ischemic stroke sequels, lacunar brain infarcts) | 1 |
| Suleman 2017 (Suleman et al., 2017) | Canada | adults over the age of 50 years | 50 | 70 (12) | 44% | 9.1(1.86) | **cognitive impairmen**t: MoCA and European Consortium Criteria | cfPWV was not associated with global cognitive decline or with individual domains of cognition | age and sex | 1 |
| Scuteri 2005 (Scuteri et al., 2005) | Italy | Older subjects referred for memory deficits | 84 | 78 (5) | 36% | 13.27 | **MMSE score** | after adjustment, cfPWV was inversely correlated with the Mini Mental State Examination score | age, sex, education, prevalent cardiovascular disease, traditional cardiovascular risk factors, medication use | 1 |
| Riba-Llena 2016 (Riba-Llena et al., 2016) | Spain | community-based population of hypertensive patients from ISSYS Study | 699 | 68.2 (5.3) | 49.9% | 10.6 (2.2) | **global cognitive function:** dementia screening test (Dementia Rating Scale second version, DRS-2) | cfPWV was inversely correlated with executive function and total scores, but after adjustment, associations were lost. | age, sex, education, antihypertension, antiplatelet and statin treatments (when appropriate), SBP and other vascular factors, cerebral small vessel disease lesions. | 4 |
| Poels 2007 (Poels et al., 2007) | Netherlands | population-based cohort from Rotterdam Study | 3714 | 72.0 (6.7) | 42.3% | 13.5 (3.0) | **Executive cognitive function:** Letter-Digit Substitution Task; **verbal fluency:** Word Fluency Test; | this study did not find an association between arterial stiffness and cognitive decline or the risk of dementia | age, sex, and education; arterial pressure, heart rate, current smoking, diabetes mellitus, body mass index, total cholesterol, high-density lipid cholesterol, and intima media thickness. | 5 |
| Palta 2019 (Palta et al., 2019) | USA | aged 67–90 years olders from ARIC Study | 3703 | 75.2 (5.0) | 40.7% | 11.22 (9.55-13.24) | **general cognitive performance;** **Memory:** delayed word recall, logical memory, and incidental learning; **executive functioning/processing speed:** Trail Making Tests, parts A and B; Digit Span Backwards; and Digit Symbol Substitution Test; **language:** semantic and phonemic fluency and Boston Naming Test. | participants in the highest quartile had statistically significantly lower cognitive factor scores in the domains of executive function/processing speed (by −0.04 z score [95% CI, −0.07 to −0.01 z score]) and general cognitive performance (by −0.09 z score [95% CI, −0.15 to −0.03 z score]; Differences in memory and language factor scores across quartiles of cfPWV were not statistically supported (P>0.05). | age, sex, race‐center, education, apolipoprotein E4, heart rate, body mass index, ever smoker, diabetes mellitus, minutes of leisure‐time physical activity, and MAP. | 4 |
| Meyer 2017 (Meyer et al., 2017) | USA | participants aged 70–89 from ARIC-NCS study | 4461 | 75.4 (5.0) | 41.2% | 11.71 (3.48) | **cognitive impairment and dementia:** based on the National Institute on Aging-Alzheimer’s and Diagnostic and Statistical Manual of Mental Disorders (DSM) 5th edition. | Among whites, those with cfPWV ≥75th percentile had a higher prevalence of mild cognitive impairment compared to participants <75th percentile (OR 1.27 (1.02, 1.56), but not in black participants. | age, sex, heart rate, education, study center (for whites only due to small numbers in blacks), apolipoprotein E genotype, smoking status, and physical activity | 4 |
| Nilsson 2017 (Nilsson et al., 2017) | Sweden | 61–85 years Swedish from MDC study | 2954 | 72.1(5.5) | 39.4% | 10.5 (2.4) | **MMSE score**; **perceptual and cognitive speed:** quick test of cognitive speed (AQT). | cfPWV was not associated with subtypes of dementia (Alzheimer’s disease, vascular dementia, mixed dementia) | age, sex and education; heart rate, MAP, weight, height, smoking, total serum cholesterol, prevalent diabetes, and blood pressure lowering and lipid-lowering drugs. | 5 |
| Nilsson 2014 (Nilsson et al., 2014) | Sweden | 61–85 years Swedish from MDC study | 2637 | 72.1 (5.6) | 39.2% | 10.5 (2.5) | **MMSE score;** **perceptual and cognitive speed:** quick test of cognitive speed (AQT). | When cfPWV >13.8 was added to the model, the linear association between continuous cfPWV and AQT disappeared (B = –0.08; P = 0.72), but cfPWV >13.8 was highly significant (B = 4.81; P = 0.004) | age, sex, and education; MAP, heart rate, smoking status, total serum cholesterol, height, weight, diabetes status, and blood-pressure-lowering and lipid-lowering drugs | 5 |
| Muela 2018 (Muela et al., 2018) | Brazil | patients with hypertension | 142 | 51.8 (11.85) | 45.1% | 7.9 (1.2) | **MMSE score; global cognitive function:** MoCA; **Language:** BNT; **Memory:** RAVLT5, RAVLT6, RAVLT7 and REY‐30;**Executive function:** VF, FAS, BDST, and TMT‐B; **Visuospatial abilities:** REY‐C and CDT; **Attention:** FDST and TMT‐A; **processing speed** DSST. | Individuals with higher PWV had poorer cognitive performance in all cognitive domains | education | 2 |
| Lim 2016 (Lim et al., 2016) | Singapore | community-dwelling older Asians from SLAS-II Study cardiac diseases | 308 | 63.0 (6.1) | 55.2% | 4.96 (2.55–14.10) | **MMSE score;** **attention:** Digit Span-Forward and Colour Trails Test ; **verbal memory:** Rey Auditory Verbal Learning Test, Story Memory and Recall; **language:** Boston Naming Test; **visuospatial ability:** Brief Visuospatial Memory Test-Revised; **executive function:** Digit Span Backward, Block Design, Colour Trails Test 2, Animal Naming. | after adjustment, cfPWV was associated with executive function(β=0.209, p = 0.026). | age, gender, education, hypertension, diabetes, dyslipidemia, smoking, BMI, and apolipoprotein E4 genotype | 5 |
| Kearney-Schwartz 2009 (Kearney-Schwartz et al., 2009) | French | elderly hypertensive patients with subjective memory complaints | 198 | 69.3 (6.2) | 50% | 11.5 (2.4) | **MMSE score;** **memory and language:** Grober-Buschke test; **visuoperceptual and visuospatial capacities:** Benton Visual Retention Test; **executive function and long-term verbal memory:** Verbal Fluency Test. | Only in male patients, PWV was the significant predictor (OR, 1.22; 95% CI, 1.00 to 1.49; P=0.05) | age, gender, diabetes, smoking, body mass index, total cholesterol, low-density lipoprotein cholesterol, triglycerides, fasting glucose, Fazekas grade, blood pressure, carotid artery structural parameters; estimated glomerular filtration rate, and left ventricular hypertrophy. | 1 |
| Hanon 2005 (Hanon et al., 2005) | French | olders>60 years with memory symptoms | 308 | 78 (8) | 36% | 12.76 (2.84) | **MMSE score**; **global cognitive function:** the cognitive efficiency profile (CEP). | after adjustment a significant relationship was observed between PWV and MMSE (β=−0.091; SE=0.028; P<0.001) and between PWV and CEP scores (β=−0.029; SE=0.009; P<0.001); For each 2 m/s increment in PWV, the adjusted OR was 1.73 (95% CI, 1.27- 2.47; P=0.001) for AD and 3.52 (95% CI, 1.87-8.05; P<0.001) for VaD. | age, gender, SBP, education level, antihypertensive therapy, presence of cardiovascular diseases | 4 |
| Geijselaers 2016 (Geijselaers et al., 2016) | Netherlands | middle age and older individuals from Maastricht Study | 725 | 60 (8) | 54.6% | 8.9 (2.1) | **free recall memory:** Verbal Learning Test; **processing speed:** Stroop Colour Word Test Part I and II, the Concept Shifting Test Part A and B, and the Letter-Digit Substitution Test; **Executive function and attention:** Stroop Colour Word Test Part III and the Concept Shifting Test Part C | cfPWV was not associated with cognitive performance | age, sex, and educational level; arterial pressure and heart rate; type 2 diabetes, body mass index, lipids, use of antihypertensive medication, estimated glomerular filtration rate, smoking behaviour, alcohol consumption, current depression, history of cardiovascular disease(s). | 5 |
| Elias 2009 (Elias et al., 2009) | USA | dementia- and stroke-free participants of the Maine-Syracuse Longitudinal Study | 409 | 61.3 (12.8) | 37.7% | 10.2 (2.8) | **visual-spatial organization and memory:** block design, object assembly, visual reproductions immediate and delayed, Hooper Visual Organization Test, and matrix reasoning; **scanning and tracking:** Trail Making Tests A and B, digit symbol substitution, and symbol search; **verbal episodic memory:** logical memory immediate and delayed and Hopkins Verbal Learning Test; **working memory:** digit span forward and backward, letter-number sequence and controlled oral word associations; **Global cognitive function:** global composition. | cfPWV (1 m/s) were inversely and significantly related to the scanning and tracking composite score (β=−.0468; SE=0.0183; P<0.01). | age, education, sex, height, weight, heart rate, brachial MAP, antihypertensive medications, reciprocal creatinine, trait anxiety, depressed mood, diabetes mellitus, cardiovascular disease, number of previous exams, race/ethnicity, total cholesterol, cigarettes per week, apolipoprotein E, homocysteine, PWV, and PWV×age | 5 |
| DuBose 2019 (Dubose et al., 2019) | USA | middle-aged/older adults from “Aging, Vascular Disease and Cognition” study | 113 | 66.6 (7.4) | 49.6% | 10.4 (2.1) | **Global cognitive performance:** Repeatable Battery for the Assessment of Neuropsychological Status (RBANS); **Processing speed and executive function performance:** Stroop Color Word Reading Test; | cfPWV (β = −.03, p = .75) alone was not associated with executive function; Greater cfPWV was associated with weaker executive function performance in the lower education group (r = −.39, p = .03), but not in the higher education group (r = .15, p = .21). | age, sex, medication, and mean arterial pressure | 3 |
| Cooper 2016 (Cooper et al., 2016) | Iceland | olders from AGES-Reykjavik Study | 1820 | 80 (5) | 40% | 13.6 (4.6) | **memory:** California Verbal Learning Test immediate and delayed recall; **processing speed:** Digit Symbol Substitution Test, Figure Comparison and the Stroop Test, Parts I (word naming) and II (color naming); **executive function:** Digits Backward and the Stroop Test and Part III (word–color interference); **global cognitive function:** Composite scores . | Higher cfPWV was related to lower memory score (P=0.002) but was not related to processing speed (P=0.97) or executive function (P=0.90) in a model that adjusted for vascular risk factors. | age, height, weight, heart rate, diabetes mellitus, previous cardiovascular disease, use of antihypertensive and lipid-lowering medication, total and high-density lipoprotein cholesterol levels, triglycerides, smoking, education level, and depressive symptoms | 4 |
| Zhong 2014 (Zhong et al., 2014) | USA | older adults from EHLS study | 1394 | 74.9 (7.2) | 43% | 11.0 (3.6) | **MMSE score;** **executive function, attention and speed:** Trail Making Test -part A and -part B (TMT-A and TMT-B, respectively); **psychomotor speed and sustained attention:** Digit Symbol Substitution Test (DSST); **memory:** Rey Auditory Verbal Learning Test (AVLT). | cfPWV >12 m/s but not the continuous cfPWV was associated with cognitive function. | age, sex, education; pulse rate, hemoglobin A1C, cardiovascular risk factors; cholesterol, hypertension, cardiovascular history, smoking and drinking, and depression symptoms. | 4 |
| Kim 2017 (Kim et al., 2017) | USA | incident hemodialysis participants from PACE study | 333 | 55 (13) | 58% | 10.0 (7.9−12.5) | **global cognitive impairment:** 3MS; **executive function:** Trail Making Tests A and B (TMTA and TMTB); | cfPWV was not independently associated with TMTA, TMTB, or 3MS after adjustment. | age, sex, race, Charlson comorbidity index, wide Range Achievement Test 4th edition reading score; SBP. | 2 |
| Dixon 2020 (Dixon et al., 2020) | Australia | older adults with subjective mood/cognitive complaints | 56 | 69.71 (8.80) | 65% | 11.65 (2.01) | **processing speed:** Trail Making Test Part A (TMT-A); **executive function:** Trail Making Test Part B (TMT-B); **response inhibition:** Delis-Kaplan Executive Functioning System Stroop task (specifically examining the Inhibition/Switching subtest of the D-KEFS Color-Word Interference Task); **verbal learning and memory:** Rey Auditory Verbal Learning Test (RAVLT); | Individuals with high cfPWV (>/=12.0m/s) had poorer performance on TMT-B, compared to low PWV (<12.0m/s), and a moderate negative correlation (r = -0.38, p = .004) between PWV and TMT-B performance. | age, MAP and 15-item Geriatric Depression Scale. | 2 |
| Karasavvidou 2018 (Karasavvidou et al., 2018) | Greece | middle‐aged chronic kidney disease (CKD) population | 151 | 57.08 (13.7) | 64.5% | 6.5 (1.87) | **MMSE score; executive and visual function:** Clock‐test | High values of cfPWV was independent determinants of cognitive decline assessed by the MMSE. | age, education, PP, Glomerular Filtration Rate, morphometric (sex, height, weight, BMI, body surface area [BSA]), blood pressure, lipids blood, inflammation, mineral metabolism and treatment. | 1 |
| Kennedy 2018 (Kennedy et al., 2018) | Australia | aged between 60-90 years olders from LIILAC trial | 102 | 77.5 (6.9) | 28% | 11.51 (1.94) | **Reaction and Decision Speed:** Simple Reaction Time (SRT), Choice Reaction Time (CRT); **Cognitive Processing:** Immediate Recognition (IRec), Congruent Stroop (CStrp); **Spatial Working Memory:** Spatial Working Memory (SWM); **Visual Processing:** Immediate Recognition (IRec), Contextual Memory (CMem), and Delayed Recognition (DRec). | aortic stiffness independently associated with Spatial Working Memory (SWM) performance | age, sex and education | 3 |

mNOS: modified Newcastle Ottawa Scale; ESRD: end-stage renal disease; SD: standard deviation; SE: standard error; PWV, pulse‐wave velocity; cfPWV, carotid‐femoral pulse‐wave velocity; SBP, systolic blood pressure; DBP, diastolic blood pressure; MAP: mean arterial pressure; PP: pulse pressure; BMI: body mass index; MMSE, Mini‐Mental State Examination; MCI, mild cognitive impairment; AD: Alzheimer’s disease; VaD: vascular dementia; OR: odds ratio; RR: relative risk; IQR: interquartile range; ^†^: age was indicated as mean (SD); ^‡^: aortic PWV was indicated as mean (SD), median (IQR) or ranged value according the expression in studies.
