# Supplementary material for: Association of Aortic Stiffness and Cognitive Decline: A Systematic Review and Meta-Analysis
Source: Front Aging Neurosci. 2021 Jun 24;13:680205. doi: 10.3389/fnagi.2021.680205 (PMC8261283; doi:10.3389/fnagi.2021.680205)
Supplement: Supplementary file 3 [file Table_2.docx]

**Table S2 characteristic of longitudinal studies**

| **First author years** | **country** | **study population** | **sample size** | **follow-up duration** | **age** ^†^ **years** | **Male %** | **PWV** ^‡^ **m/s** | **outcomes and cognition test** | **results** | **adjusted variables** | **m NOS** |
| --- | --- | --- | --- | --- | --- | --- | --- | --- | --- | --- | --- |
| Zeki 2013 (Zeki Al Hazzouri et al., 2013) | USA | community-dwelling elders from Health ABC study | 2488 | over 9 years | 74.2 (2.9) | 47.7% | middle tertile: 6.5-6.9 | **cognitive impairment:** Modified Mini-Mental Status Exam (3MS). | In fully adjusted models, the odds of cognitive impairment after 9 years of follow-up was 40% greater for subjects with middle cfPWV (odds ratio [OR], 1.40; 95% CI, 1.03–1.92) and 59% greater for subjects with high PWV (OR, 1.59; 95% CI, 1.16–2.18), compared with low cfPWV. | race/ethnicity, sex, education, apolipoprotein E4, and traditional cardiovascular risk factors, such as BMI, type2 diabetes mellitus, hypertension, and mean arterial blood pressure | 6 |
| Araghi 2019 (Araghi et al., 2019) | British | London-based British civil servants | 3828 | mean 7.3 years | 65.3 (5.7) | 74.8% | middle third: 7.41–8.91 | **Memory:** 20-word list; Reasoning:AH4-I (Alice Heim 4-I); **Verbal Fluency:** S words and animal word; **global cognitive score:** composite scores; **MMSE score;** | Accelerated 7-year cognitive decline was observed among individuals with the highest cfPWV [difference in 7-year cognitive change for highest third versus lowest third cf PWV: − 0.06, 95% CI − 0.11, − 0.01, P < 0.01]. Higher aortic stiffness was associated with faster cognitive decline. | age, sex, ethnicity and employment. | 3 |
| Watson 2011 (Watson et al., 2011) | USA | well-functioning older adults from Health ABC study | 406 | over 7 years | 73.1 (2.7) | 47.0% | 8.86 (3.89) | **global cognitive function:** Modified Mini-Mental Status Exam (3MS); **verbal learning and memory:** Buschke Selective Reminding Test (SRT); **psychomotor speed:** Boxes and Digit Copying (BDC) tests; **perceptual speed:** Pattern and Letter Comparison (PLC) tests. | After adjustment, each 1 SD higher cfPWV (389 cm/s) was associated with poorer cognitive function: −0.11 SD for global function (SE = 0.04, p < .01), −0.09 SD for psychomotor speed (SE = 0.04, p = .03), and −0.12 SD for perceptual speed (SE = 0.04, p < .01). | age, sex, race, education, and clinic site; body mass index, mean arterial pressure, cholesterol, heart rate, smoking status, physical activity, depressive symptoms, prevalent hypertension, coronary heart disease, cerebrovascular disease, and diabetes mellitus | 8 |
| Waldstein 2008 (Waldstein et al., 2008) | USA | nondemented, stroke-free persons from BLSA study | 582 | over 11 years | 54.3(17.1) | 44.3% | 7.1 (2.65) | **Attention and concentration:** Digits Forward and Digits Backward portions of the Wechsler Adult Intelligence Scale-Revised; **verbal learning and memory:** California Verbal Learning Test (CVLT); **nonverbal memory:** Benton Visual Retention Test (BVRT); **attention, perceptuo-motor speed, visuomotor scanning, and mental flexibility (an executive function):** The Trail Making Test Part A and Part B; **phonetic and semantic association fluency:** Letter Fluency and Category Fluency, confrontation naming: Boston Naming Test; | Persons with higher baseline cfPWV exhibited prospective decline on tests of verbal learning and delayed recall, nonverbal memory, and a cognitive screening measure (P<0.05). | age, education, depression scores, MAP, heart rate, BMI, and total cholesterol levels were treated as continuous covariates, and sex, smoking, alcohol use, antihypertensive medications, and cardiovascular comorbidities were treated as categorical covariates | 6 |
| Watfa 2015 (Watfa et al., 2015) | French and Italian | individuals older than 80 years from PARTAGE study | 682 | 2 years | 87.5 (4.7) | 19.0% | 14.3 (4.9) | **Cognitive decline:** MMSE; | Decliners were more likely to have higher baseline PWV values; cfPWV was higher compared with nondecliners after adjust; high cfPWV was independent factors for MMSE decline. | baseline MMSE, MBP, heart rate, BMI, age, education level, and Activities of daily living scale. | 4 |
| Pase 2016 (PaseBeiser et al., 2016) | USA | adults ≥60 years from FHS Offspring cohort | 1101 | over 10 years | 69 (6) | 46.0% | 10.7 (9.0-13.2) | **cognitive impairment, dementia and AD:** in accordance with the Diagnostic and Statistical Manual of Mental Disorders, 4th edition and National Institute of Neurological and Communicative Disorders and Stroke and the AD and Related Disorders Association for definite, probable, or possible AD. | After adjustment for age and sex, higher continuous aortic stiffness predicted an increased risk of mild cognitive impairment (hazard ratio, 1.40 [95% confidence interval, 1.13–1.73]), all-cause dementia (hazard ratio, 1.45 [95% confidence interval, 1.13–1.87]), and Alzheimer disease (hazard ratio, 1.41 [95% confidence interval, 1.06–1.87]). | age at baseline and sex; education and apolipoprotein E4 ε4 allele status, MAP, prevalent diabetes mellitus, and high-density lipoprotein-cholesterol; prevalent atrial fibrillation, current smoking, prevalent cardiovascular disease, heart rate, total cholesterol, depressive symptoms, central adiposity, and treatment for hypertension. | 8 |
| Tsao 2016 (Tsao et al., 2016) | USA | Stroke- and dementia-free participants from FHS Offspring Study | 1223 | mean 6.4 years | 61 (9) | 44.0% | 9.0 (7.6-10.9) | **executive function:** Trail Making Test Part B minus Part A (Trails B-A) score; **abstract reasoning:** similarities; | Higher cfPWV was associated with an increase in ΔTrails B-A (p = 0.019); The small decline seen in the relation of cfPWV with ΔSimilarities was not statistically significant, but aged 65 years or older demonstrated a larger magnitude of effect than younger. | age, sex, and MAP, with the addition of education and depression scale; time interval between baseline and neurocognitive testing, lipids, log-homocysteine, plus indicators for diabetes, atrial fibrillation, smoking, antihypertensive treatment, prevalent cardiovascular disease, apolipoprotein E4 ε4 allele, and fourth quartile of waist-hip ratio. | 6 |
| Scuteri 2013 (Scuteri et al., 2013) | Italy | cognitive normal but with complaints of memory loss individuals | 105 | medium 15 month | 77.2 (4.7) | 24.0% | 13.5 (2.2) | **Cognitive impairment:** MMSE . | The presence of PWV values in the upper quartile (≥14.2m/s) was accompanied by a 4-fold risk for developing cognitive impairment. | education, age, gender, and traditional caidiovascular risk factors (blood pressure, lipids, adiposity, blood glucose and diabetes) | 4 |
| Scuteri 2007 (Scuteri et al., 2007) | Italy | Older individuals with complaints of memory loss | 102 | median 12 months | 79 (6) | 30.4% | 13.5 (2.2) | **cognitive impairment:** MMSE | PWV was the single strongest predictor of cognitive decline, explaining 15.2% of the total variance (each 1 m/s increase in PWV was associated with a 0.74 per-year decrease of MMSE score, P < 0.001) | age, gender, education, body mass index, current smoking status, SBP, DBP, low-density lipoprotein and high-density lipoprotein cholesterol, fasting blood glucose, serum creatinine, baseline MMSE, and brain imaging | 4 |
| Rouch 2018 (Rouch et al., 2018) | French | subjects with memory complaint and with a diagnosis of MCI | 404 | mean 4.5 years | 75.2 (7.0) | 34.4% | 11.8 (2.2) | **Dementia:** Diagnostic and Statistical Manual of Mental Disorders, fourth revision. | higher baseline PWV independently associated with greater risk of conversion from MCI to dementia (1-SD increase of PWV: HR, 1.33; 95% CI, 1.04–1.71; P=0.02). | age, sex, educational level, SBP, cardiovascular diseases, body mass index, calcium channel blockers intake, MMSE, and apolipoprotein E4 ε4 allele status | 6 |
| Poels 2007 (Poels et al., 2007) | Netherlands | population-based cohort from Rotterdam Study | 2767 | mean 4.4 years | 70.7 (6.0) | 41.9% | 13.2 (2.9) | **MMSE score; Executive cognitive function:** Letter-Digit Substitution Task( an abbreviated Stroop Test and the Word Fluency Test); **dementia and subtypes of dementia:** internationally accepted criteria for dementia, Alzheimer disease (AD) (NINCDS-ADRDA), and vascular dementia (NINDS-AIREN). | This study did not find an association between arterial stiffness and cognitive decline or the risk of dementia. | age, sex, and education; arterial pressure, heart rate, current smoking, diabetes mellitus, body mass index, total cholesterol, high-density lipid cholesterol, and intima media thickness. | 6 |
| Nilsson 2017 (Nilsson et al., 2017) | Sweden | 61–85 years Swedish from MDC study | 2954 | medium 4.6 years | 72.1 (5.5) | 39.4% | 10.5 (2.4) | **Dementia:** Diagnostic and Statistical Manual of Mental Disorders, 5th edition. | cfPWV was not associated with subtypes of dementia (Alzheimer’s disease, vascular dementia, mixed dementia). | age, sex and education; heart rate, MAP, weight, height, smoking, total serum cholesterol, prevalent diabetes, and blood pressure lowering and lipid-lowering drugs. | 6 |
| Menezes 2019 (Menezes et al., 2019) | Brazil | middle‐aged and older adults from from ELSA‐Brasil study | 6927 | average 3.8 years | 58.8 (5.9) | 45.0% | 9.9 (1.9) | **Memory tests:** test battery Consortium to Establish a Registry for Alzheimer’s Disease; **Verbal fluency tests:** Semantic and Phonemic Verbal Fluency Test scores; **Trail test (version B).** | increase in cfPWV is associated with a more pronounced decrease in cognitive scores as the time interval between the visits increased. | sex, schooling level, smoking, consumption of alcoholic beverages, physical activity, diabetes mellitus, cardiovascular disease, total cholesterol/high‐density lipoprotein cholesterol ratio, use of lipid‐lowering drugs, use of antihypertensive drugs, weight, height, and mean heart rate | 6 |
| Hajjar 2016 (Hajjar et al., 2016) | USA | healthy and relatively young population from Health Discovery and Well Being cohort | 591 | range 1-4 years | 48.8 (9.72) | 32.0% | 7.2 (1.46) | **executive, memory, and working memory:** Delayed Memory Recall, Memory Recognition, Mental Flexibility, SPOTING The Symbol, Digit Symbol Substitution Test, Digit Span Forward, Digit Span Backwards, Executive Function Test, Visual-Spatial Memory, Visual Spatial Short Term Recall, Pattern Recall, Pattern Recall-Delayed, Pattern Recognition, Focused Attention, Sustained Attention. | Higher cfPWV was associated with a steeper decline in executive (P=0.0002), memory (P=0.05), and working memory (P=0.02) scores after adjusting; PWV explained the association between hypertension and executive function. | baseline cognitive score, age, sex, race, education, and body mass index | 6 |
| Cui 2018 (Cui et al., 2018) | USA | olders who were not demented at baseline from CHS-CS Study | 356 | over 15 years | 77.8 (3.8) | 41.0% | 8.2 (6.7-10.0) | **Dementia:** at leat two cognitive domains deficit of sufficient severity to affect subjects’ activities of daily living function | cfPWV was significantly associated with increased risk of dementia. | age, sex; education, race, apolipoprotein E4, diabetes, BMI, MAP; anti-hypertensive medication | 7 |
| Kim 2017 (Kim et al., 2017) | USA | incident hemodialysis participants from PACE study initiation | 135 | 1 year | 55 (13) | 57.0% | 9.3 (7.7-12.0) | **global cognitive impairment:** 3MS; **executive function:** Trail Making Tests A and B (TMTA and TMTB); | cfPWV was not independently associated with TMTA, TMTB, or 3MS after adjustment. | age, sex, race, Charlson comorbidity index, Wide Range Achievement Test 4th edition reading score; systolic blood pressure. | 3 |

mNOS: modified Newcastle Ottawa Scale; SD: standard deviation; PWV, pulse‐wave velocity; cfPWV, carotid‐femoral pulse‐wave velocity; SBP, systolic blood pressure; DBP, diastolic blood pressure; MAP: mean arterial pressure; BMI: body mass index; MMSE, Mini‐Mental State Examination; MCI, mild cognitive impairment; AD: Alzheimer’s disease; OR, odds ratio; RR, relative risk; ^†^: age was indicated as mean (SD); ^‡^: aortic PWV was indicated as mean (SD), median (IQR, interquartile range) or ranged value according the expression in studies.
