# Supplementary material for: Association of Aortic Stiffness and Cognitive Decline: A Systematic Review and Meta-Analysis
Source: Front Aging Neurosci. 2021 Jun 24;13:680205. doi: 10.3389/fnagi.2021.680205 (PMC8261283; doi:10.3389/fnagi.2021.680205)
Supplement: Supplementary file 4 [file Table_3.docx]

**Table S3. Heterogeneity and publication bias of each sensitive analysis in association between aortic PWV and cognitive function**

|  | **assessed items** |  | **attention** | **Executive Function** | **global cognitive function** | **Memory** | **processing speed** | **Visuospatial ability** |
| --- | --- | --- | --- | --- | --- | --- | --- | --- |
| **analysis 1** | Number of studies | | 9 | 13 | 6 | 14 | 13 | 5 |
|  | Heterogeneity | p (Q test) | 0 | 0 | 0 | 0 | 0 | 0 |
|  |  | I-squared | 95.57 | 88.05 | 77.77 | 73.27 | 88.49 | 93.59 |
|  | Egger's regression | intercept | -4.53 | -1.56 | -0.24 | -2.37 | -1.65 | -11.47 |
|  |  | p (2-tails) | 0.13 | 0.29 | 0.95 | 0.01 | 0.28 | 0.11 |
| **analysis 2** | Number of studies | | 7 | 11 | 6 | 12 | 11 | 5 |
|  | Heterogeneity | p (Q test) | 0.01 | 0 | 0 | 0.02 | 0.01 | 0 |
|  |  | I-squared | 66.41 | 82.16 | 77.77 | 51.41 | 56 | 93.59 |
|  | Egger's regression | intercept | -0.89 | -0.12 | -0.24 | -1.52 | 0.39 | -11.47 |
|  |  | p (2-tails) | 0.56 | 0.93 | 0.95 | 0.08 | 0.68 | 0.11 |
| **analysis 3** | Number of studies | | 5 | 9 | 4 | 10 | 10 | 4 |
|  | Heterogeneity | p (Q test) | 0.23 | 0 | 0 | 0.4 | 0.08 | 0 |
|  |  | I-squared | 28.53 | 82.94 | 84.54 | 4.64 | 41.74 | 94.9 |
|  | Egger's regression | intercept | 0.7 | 0.72 | 2.56 | -0.79 | 1.14 | -13.68 |
|  |  | p (2-tails) | 0.6 | 0.67 | 0.7 | 0.29 | 0.18 | 0.19 |
| **analysis 4** | Number of studies | | 4 | 8 | 3 | 9 | 9 | 4 |
|  | Heterogeneity | p (Q test) | 0.14 | 0 | 0.18 | 0.35 | 0.05 | 0 |
|  |  | I-squared | 45.35 | 81.91 | 40.97 | 10.3 | 47.97 | 94.9 |
|  | Egger's regression | intercept | 1.97 | -0.29 | 3.61 | -0.74 | 1.39 | -13.68 |
|  |  | p (2-tails) | 0.38 | 0.88 | 0.07 | 0.43 | 0.17 | 0.19 |

Analysis 1: Included all the eligible studies; Analysis 2: Excluded studies with participant of chronical kidney disease; Analysis 3: Further excluded studies with participants of hypertension based on analysis 2; Analysis 4: Further excluded studies with participant complaining loss of memory based on analysis 3, and just included studies with participants from general middle/older adults.
