# Supplementary material for: Association of Aortic Stiffness and Cognitive Decline: A Systematic Review and Meta-Analysis
Source: Front Aging Neurosci. 2021 Jun 24;13:680205. doi: 10.3389/fnagi.2021.680205 (PMC8261283; doi:10.3389/fnagi.2021.680205)
Supplement: Supplementary file 5 [file Table_4.docx]

**Table S4. Cross sectional analysis of association between aortic PWV and MMSE**

| **results** | **Analysis 1** | **Analysis 2** | **Subgroup analysis #** |
| --- | --- | --- | --- |
| number of studies | 11 | 4 | 3 |
| participants | 9034 | 8053 | 448 |
| r (95% CI) | -0.11 (-0.15, -0.07)* | -0.02 (-0.041, 0.002) | -0.27 (-0.35, -0.18)* |
| Q test (p value) | 0 | 0.6 | 0.995 |
| I-squared (%) | 76.4 | 0 | 0 |
| Egger's regression (p value) | 0.002 | 0.37 | 0.15 |

Analysis 1: Included all the eligible studies; Analysis 2: Excluded studies with participant of special disease (chronical kidney disease, hypertension and complaint of memory loss), and just included studies with participants from general middle/older adults; #: subgroup analysis for three studies including participants with complaining loss of memory; *: p<0.05
