# Supplementary material for: Association of Aortic Stiffness and Cognitive Decline: A Systematic Review and Meta-Analysis
Source: Front Aging Neurosci. 2021 Jun 24;13:680205. doi: 10.3389/fnagi.2021.680205 (PMC8261283; doi:10.3389/fnagi.2021.680205)
Supplement: Supplementary file 6 [file Table_5.docx]

**Table S5. Meta-regression analysis of association between aortic PWV and cognitive function, cognitive impairment and dementia for all included studies.**

| **outcomes** | **age** | | | **male (%)** | | | **MBP** | | | **lower education level (%)** | | |
| --- | --- | --- | --- | --- | --- | --- | --- | --- | --- | --- | --- | --- |
|  | **n** | **β (SE)** | **p value** | **n** | **β (SE)** | **p value** | **n** | **β (SE)** | **p value** | **n** | **β (SE)** | **p value** |
| **Cross-sectional study** |  |  |  |  |  |  |  |  |  |  |  |  |
| attention | 9 | 0.016  (0.011) | 0.151 | 9 | -0.031  (0.007) | <0.001 | 7 | -0.038  (0.021) | 0.066 | 5 | -0.017  (0.011) | 0.122 |
| global cognitive function | 6 | 0  (-0.0062) | 0.998 | 6 | 0.021  (0.005) | <0.001 | 5 | -0.02  (0.012) | 0.146 | 4 | -0.01  (0.003) | <0.001 |
| memory | 14 | 0.002  (0.002) | 0.286 | 14 | -0.008  (0.0031) | 0.009 | 10 | -0.011  (0.005) | 0.025 | 8 | -0.002  (0.001) | 0.243 |
| procession speed | 13 | 0.009  (0.004) | 0.033 | 13 | -0.016  (0.004) | <0.001 | 10 | -0.031  (0.009) | <0.001 | 7 | -0.005  (0.003) | 0.097 |
| MMSE score | 11 | 0.002  (0.003) | 0.61 | 11 | -0.002  (0.003) | 0.581 | 8 | 0.011  (0.01) | 0.299 | 5 | -0.001  (0.002) | 0.72 |
| **longitudinal study**  **(for categorical cfPWV)** | | |  |  |  |  |  |  |  |  |  |  |
| cognitive impairment | 6 | 0.032  (0.011) | 0.004 | 6 | -0.013  (0.011) | 0.2 | 5 | 0.034  (0.033) | 0.31 | 4 | 0.002  (0.01) | 0.798 |
| dementia | 3 | na | na | 3 | na | na | 3 | na | na | 3 | na | na |
| **longitudinal study**  **(for continuous cfPWV)** | | |  |  |  |  |  |  |  |  |  |  |
| cognitive impairment | 6 | 0  (0.002) | 0.897 | 6 | 0  (0.001) | 0.969 | 5 | -0.008  (0.005) | 0.095 | 6 | 0  (0.001) | 0.626 |
| dementia | 5 | -0.015  (0.029) | 0.6 | 5 | 0.028  (0.018) | 0.12 | 5 | -0.022  (0.013) | 0.105 | 4 | -0.004  (0.004) | 0.27 |

MBP: mean blood pressure; SE: standard error; na: not available.
