# Supplementary material for: Association of Aortic Stiffness and Cognitive Decline: A Systematic Review and Meta-Analysis
Source: Front Aging Neurosci. 2021 Jun 24;13:680205. doi: 10.3389/fnagi.2021.680205 (PMC8261283; doi:10.3389/fnagi.2021.680205)
Supplement: Supplementary file 7 [file Table_6.docx]

**Table S6. Meta-regression analysis of association between aortic PWV and cognitive function, cognitive impairment and dementia for studies that excluded those of participants with specific disease.**

| **outcomes** | **age** | | | **male (%)** | | | **MBP** | | | **lower education level (%)** | | |
| --- | --- | --- | --- | --- | --- | --- | --- | --- | --- | --- | --- | --- |
|  | **n** | **β (SE)** | **p value** | **n** | **β (SE)** | **p value** | **n** | **β (SE)** | **p value** | **n** | **β (SE)** | **p value** |
| **Cross-sectional study** |  |  |  |  |  |  |  |  |  |  |  |  |
| attention | 3 | na | na | 3 | na | na | 1 | na | na | 2 | na | na |
| global cognitive function | 4 | -0.02  (0.017) | 0.272 | 4 | 0.023  (0.007) | <0.001 | 3 | na | na | 4 | -0.01  (0.003) | <0.001 |
| memory | 9 | -0.002  (0.001) | 0.065 | 9 | -0.002  (0.002) | 0.414 | 6 | -0.002  (0.001) | 0.094 | 7 | -0.0004  (0.0002) | 0.482 |
| procession speed | 9 | 0  (0.002) | 0.939 | 9 | -0.003  (0.003) | 0.31 | 7 | -0.003  (0.006) | 0.679 | 6 | -0.001  (0.001) | 0.516 |
| MMSE score | 4 | 0.006  (0.005) | 0.255 | 4 | -0.005  (0.004) | 0.202 | 2 | na | na | 4 | 0.0004  (0.0005) | 0.414 |
| **longitudinal study**  **(for categorical cfPWV)** | | |  |  |  |  |  |  |  |  |  |  |
| cognitive impairment | 5 | 0.03  (0.01) | 0.003 | 5 | -0.007  (0.011) | 0.549 | 4 | 0.012  (0.037) | 0.744 | 4 | 0.002  (0.01) | 0.798 |
| dementia | 2 | na | na | 2 | na | na | 2 | na | na | 2 | na | na |
| **longitudinal study**  **(for continuous cfPWV)** | | |  |  |  |  |  |  |  |  |  |  |
| cognitive impairment | 6 | 0  (0.002) | 0.897 | 6 | 0  (0.001) | 0.969 | 5 | -0.008  (0.005) | 0.095 | 6 | 0  (0.001) | 0.626 |
| dementia | 4 | -0.008  (0.036) | 0.821 | 4 | 0.03  (0.025) | 0.227 | 4 | -0.036  (0.011) | 0.001 | 3 | na | na |

MBP: mean blood pressure; SE: standard error; na: not available.
